# Exposure to secondhand smoke and physical disabilities in non-smokers: A national cross-sectional study with cotinine measurements from NHANES 2013–2018

**DOI:** 10.18332/tid/200546

**Published:** 2025-02-21

**Authors:** Jiahui He, Zhounan Wu, Yuhang Liang, Jinshen He

**Affiliations:** 1 Department of Orthopaedic Surgery, Third Xiangya Hospital of Central South University, Changsha, People’s Republic of China; 2 Xiangya School of Medicine, Central South University, Changsha, People’s Republic of China

**Keywords:** secondhand smoke, cotinine, disability, NHANES, cross-sectional

## Abstract

**INTRODUCTION:**

Secondhand smoke (SHS) exposure is a significant health risk, but current research often overlooks its broader impact on functional impairments in the general public.

**METHODS:**

This study utilized serum cotinine levels (SCL) from the 2013–2018 NHANES dataset to investigate physical disabilities associated with SHS exposure. SCL represents the combined concentration of cotinine and hydroxycotinine. The physical disabilities assessed include difficulties with hearing, vision, concentration, walking, dressing or bathing independently, and running errands alone. Logistic regression was applied to evaluate the relationship between SCL and physical disabilities in adults, with stratified analyses by age, gender, and race. A p<0.05 was considered significant.

**RESULTS:**

Logistic regression analyses showed that SHS exposure was significantly associated with walking difficulty. Participants in the highest quartile of SCL (Q4) had significantly higher odds of walking difficulty compared to those in the lowest quartile (Q4 vs Q1, AOR=2.03; 95% CI: 1.24–3.31, p-trend=0.010). Higher hydroxycotinine were associated with increased walking difficulty (AOR=1.48; 95% CI: 1.06–2.08, p=0.030). Individuals in the highest quartile of hydroxycotinine (Q4) faced more difficulty running errands (AOR=2.09; 95% CI: 1.13–3.88, p-trend=0.036). Among males, the highest quartiles of cotinine and hydroxycotinine were more strongly associated with walking difficulty than in females (cotinine: AOR=2.92 vs 1.49; hydroxycotinine: AOR=3.23 vs 1.78). In adults aged ≥60 years, higher SCL, cotinine, and hydroxycotinine levels were significantly associated with walking difficulty (SCL, AOR=1.58; 95% CI: 1.24–2.02); cotinine, AOR=1.80; 95% CI: 1.21–2.67; hydroxycotinine, AOR=4.57; 95% CI: 1.92–10.89). An 'L'-shaped association was observed for ln(hydroxycotinine) and walking difficulty, with a significant association beyond -1.306 (AOR=2.57; 95% CI: 1.33–4.96, p=0.005).

**CONCLUSIONS:**

Higher SHS is significantly associated with various physical disabilities, especially in men and older adults.

## INTRODUCTION

Tobacco use is prevalent worldwide, with approximately 1.3 billion smokers, contributing to >8 million deaths annually, of which >7 million are due to direct smoking and 1.2 million result from secondhand smoke (SHS) exposure^1,2^. SHS contains >4000 chemical compounds, including nicotine and carbon monoxide, which stimulate the cardiovascular system, as well as carcinogens like polycyclic aromatic hydrocarbons and volatile substances such as formaldehyde^3-5^. Prolonged SHS exposure is strongly associated with increased risks of cardiovascular diseases (CVD), various cancers, and overall mortality^3,6^. Since 1964, extensive research has focused on SHS, highlighting the significant public health risks posed by SHS^7^.

Most studies to date focus on specific diseases linked to SHS exposure. For instance, a 2023 cross-sectional study in South Korea found a significant association between SHS exposure and allergic diseases in adolescents^8^. Another study from the same year linked SHS exposure to myopia development^9^. However, beyond disease outcomes, it is critical to examine how SHS affects daily functioning. Due to limited health literacy, the general public is more likely to notice discomfort or functional impairments from SHS rather than the underlying pathological changes^10^. Understanding these functional impairments can enhance public awareness and promote healthier lifestyle choices^10^. Recent research has begun to address this gap, exploring how health risks like SHS impact daily life. For instance, a study in 2022 found that habitual smoking could significantly limit the daily living activities of the elderly; in the same year, a Mendelian randomization study reported that smoking behavior was a potential risk factor for impaired physical function among the elderly in the UK^11,12^. These findings highlight the importance of improving patients’ quality of life. However, there remains a significant gap in research on the relationship between SHS and functional impairments. In light of this, our research on passive smoking and functional impairments can provide support for the development and implementation of smoke-free policies. This will not only help raise public awareness of healthy lifestyles but also reduce functional impairment issues caused by smoking, ultimately promoting the overall health and well-being of society.

This study uses data from the 2013–2018 National Health and Nutrition Examination Survey (NHANES) to explore the impact of SHS exposure on physical disabilities in adults. Serum cotinine levels (SCL) are employed as a biomarker to quantify SHS exposure, enabling an evaluation of its broader public health implications. By leveraging NHANES’ extensive health assessments and detailed questionnaires, this research aims to address significant gaps in the current literature, testing the hypothesis that SHS exposure is strongly associated with multiple physical disabilities in adults, highlighting its clinical and public health significance.

## METHODS

### Study population

This is a secondary data analysis of cross sectional data from the 2013–2018 NHANES, managed by the Centers for Disease Control and Prevention (CDC)^13^. Participants aged 20 years and older were included in the study, which received approval from the National Center for Health Statistics Ethics Review Board. The initial cohort comprised 29400 individuals. Participants aged ≥20 years who were former or current smokers – defined as those who reported having smoked at least 100 cigarettes in their lifetime or whose serum cotinine levels were >10 ng/mL – were excluded, along with individuals whose cotinine or hydroxycotinine concentrations were below the detection limit (n=24771)^14^. Additionally, those with missing outcome variable data (n=2113) or incomplete information on covariates, including body mass index (BMI), education level, marital status, diabetes, hypertension, alcohol consumption, and cardiovascular diseases (CVD) (n=346) were excluded. The final analytic sample consisted of 2170 participants.

### Definition of exposure variables

SCL is defined as the sum of serum cotinine and hydroxycotinine concentrations. For this analysis, SCL was treated as a continuous variable, utilizing data from the 2013–2014, 2015–2016, and 2017–2018 NHANES cycles. Both cotinine and hydroxycotinine are two nicotine-derived metabolites found in SHS. Cotinine shows high specificity, while hydroxycotinine is a more sensitive biomarker for low-level exposure. Due to cotinine’s relatively short half-life (15–20 h), it may be a suitable biomarker for short-term SHS exposure^15^. Hydroxycotinine is the main metabolite of cotinine. In plasma, the concentration of cotinine is usually higher than that of hydroxycotinine. Therefore, the determination of both cotinine and hydroxycotinine may provide a more sensitive measure of SHS exposure^16^. Serum concentrations of both cotinine and hydroxycotinine were measured in units of nmol/L, with a conversion factor of 5.67516 applied to standardize the units^17^. Non-smokers in this study were classified based on two criteria: 1) SCL <10 ng/mL; and 2) a negative response to the question, ‘Have you smoked at least 100 cigarettes in your lifetime?’^14,18,19^. Given the right-skewed distributions of SCL, cotinine, and hydroxycotinine, natural log (ln) transformations were applied when conducting smooth curve fitting to assess their associations with health outcomes. The lower limit of detection (LLOD) for cotinine and hydroxycotinine in serum is 0.015ng/mL, Participants with serum cotinine concentrations below this threshold were excluded from the study, in accordance with established protocols in previous research^14^. For analysis, the SCL (ng/mL) were categorized into quartiles as follows: <0.076 (Q1), 0.076–0.145 (Q2), 0.146–0.458 (Q3), and >0.458 (Q4). Similarly, hydroxycotinine levels (ng/mL) were divided into: <0.023 (Q1), 0.023–0.041 (Q2), 0.042–0.111 (Q3), and >0.112 (Q4). For cotinine (ng/mL), the quartile ranges were: <0.049 (Q1), 0.049–0.100 (Q2), 0.101–0.334 (Q3), and >0.334 (Q4). Further details on SCL can be found at the NHANES website (https://wwwn.cdc.gov/Nchs/Nhanes/2013-2014/COT_H.htm).

### Definition of outcome variables

The physical disability questionnaire consisted of six questions regarding whether or not the individual had physical, mental, or emotional conditions that caused serious difficulty with hearing (including deafness), seeing (including blindness), attention deficit hyperactivity disorder (ADHD), dressing or bathing independently, or running errands alone, such as visiting a doctor’s office or shopping. Disability status was categorized as either 'none' or 'any' disabilities, with an affirmative response to any of the six questions indicating the presence of a disability. Detailed versions of the questionnaire are accessible on the NHANES website (https://www.cdc.gov/nchs/nhanes/index.htm).

### Covariates

**Figure 1:**
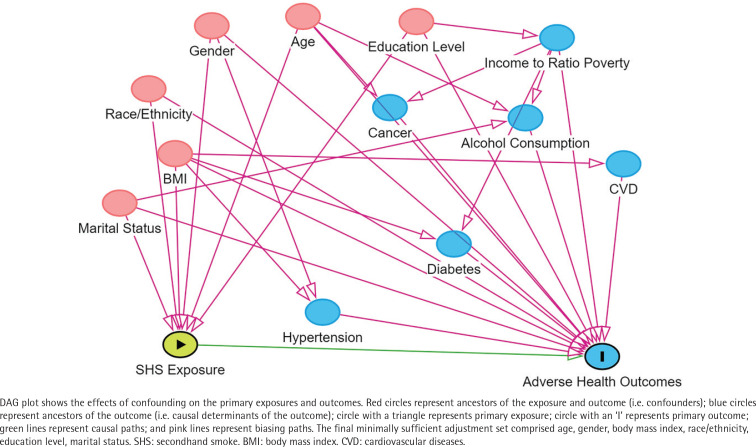
Directed acyclic graph (DAG)

In this study, confounding factors were categorized into three main groups: basic demographic variables (including gender, age, race, and BMI, health behaviors such as alcohol consumption, and health conditions including diabetes, hypertension, cancer, and CVD)^20^. The study includes categorical variables: gender, race/ethnicity (Non-Hispanic White, Non-Hispanic African American, Other), poverty income ratio (PIR), alcohol consumption, data release cycle, education level, marital status, hypertension status, diabetes, cancer, and CVD. Continuous covariate is age. BMI (kg/m^2^) was classified into two categories: <30 and ≥30. To handle missing and categorical issues with alcohol consumption, we grouped ‘refused to answer’, ‘missing data’, and ‘don’t know’ into one category. Following other studies, heavy drinkers were defined as females consuming >1 and males >2 standard drinks per day on average in the past year, with missing responses grouped as ‘unknown’^21^. Hypertension, diabetes, and CVD diagnoses were based on self-reported physician diagnoses. Participants who self-reported a physician-confirmed diagnosis of heart failure, angina, coronary heart disease, heart attack, or stroke, as confirmed by a physician, were categorized as having CVD^22^. Finally, other categorical variables included poverty income ratio (PIR) (grouped into: <1.3, 1.3–3.5, and >3.5), with missing data on PIR similarly categorized into a single ‘unknown’ category. Education level was categorized into three groups: less than high school, high school graduate, and greater than high school. Marital status was classified as either married or living with a partner, or living alone; and data release cycle as predefined. For more details, refer to NHANES (https://wwwn.cdc.gov/nchs/nhanes/). Covariate selection was guided by previous studies^23,24^ and directed acyclic graph (DAG) analysis using DAGitty v3.1 (Figure 1).

### Statistical analysis

Chi-square tests and linear regression were used to assess group differences in serum cotinine levels characteristics. Multiple logistic regression examined the relationship between exposure variables (SCL, cotinine, hydroxycotinine) and binary outcomes, with quartile analyses. Three models were applied: Model 1 (unadjusted), Model 2 (adjusted for gender and age), and Model 3 (adjusted for age, gender, body mass index, race/ethnicity, data release cycle, education level, marital status, income to poverty ratio, alcohol use, hypertension, diabetes, cancer, and CVD). In stratified analyses, we stratified by age (20–39, 40–59 or ≥60 years), sex (male or female), race/ethnicity (non-Hispanic White, non-Hispanic African American or Other) to explore heterogeneity in associations. A significance criterion was established at p<0.05. Variance inflation factor (VIF) testing assessed multicollinearity among confounders^25^. The weighted RCS curve was not only used to explore the relationship between ln-transformed hydroxycotinine and physical disabilities, but also to explore the associations between ln-transformed SCL, cotinine, hydroxycotinine and physical disabilities in different age groups. When nonlinear correlations were found, threshold effects were calculated and each interval was fitted using a two-stage linear regression model, also known as a segmented regression model. A power analysis, conducted using G*Power version 3.1.9.7, indicated that a sample size of 195 was sufficient for robust statistical analysis. Our final sample size of 2170 met this requirement. Chi-squared tests, and multiple logistic regressions were weighted according to NHANES guidelines to ensure representativeness of the US population^26^. Further details on weighting can be found at: https://wwwn.cdc.gov/nchs/nhanes/tutorials/weighting.aspx.

Data were analyzed using EmpowerStats (version 4.0), R (version 4.3.1), and G*Power version 3.1.9.7. A two-sided p<0.05 was considered a statistically significant difference.

## RESULTS

### Participant characteristics

**Figure 2:**
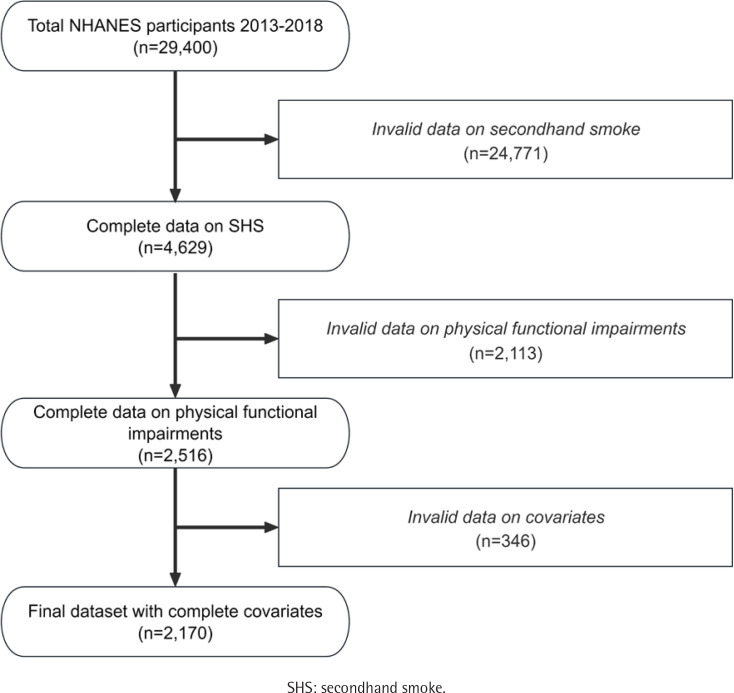
Flow chart of sample selection from NHANES 2013–2018

A detailed study design can be found in Figure 2. Table 1 and Supplementary file Table 1 present baseline cohort characteristics (mean age: 43.7 years; 37.9% male; 57.2% Non-Hispanic White). A total of 2170 participants were recruited. Significant differences were observed in the baseline characteristics across serum cotinine level subgroups. Compared with the other subgroups, participants with serum cotinine levels >0.458 ng/mL (Quartile 4) were more likely to be younger, non-Hispanic African American, married or living with a partner, of lower education, and from low-income backgrounds. VIFs for all covariates ranged from 1.020 to 1.587, indicating no significant multicollinearity (Supplementary file Table 2).

**Table 1:** The characteristics of participants (weighted)

*Characteristics*	Total* %	*Total %*	*Quartiles (ng/mL Serum cotinine level)*				
			*Q1* *%*	*Q2* *%*	*Q3* *%*	*Q4* *%*	*p ^b^*
**Total,** n	2170	2170	543	537	547	543	
**Age** (years), mean ± 95%CI	46.15 ± 0.78	43.66 ± 1.07	48.35 ± 2.07	44.64 ± 1.89	42.78 ± 1.72	38.09 ± 2.27	<0.001
**Health status**							
Serum cotinine level (ng/mL), mean ± 95%CI	0.71 ± 0.07	0.69 ± 0.07	0.05 ± 0.00	0.11 ± 0.00	0.26 ± 0.01	2.45 ± 0.23	<0.001
Cotinine (ng/mL), mean ± 95%CI	0.52 ± 0.05	0.49 ± 0.05	0.03 ± 0.00	0.07 ± 0.00	0.19 ± 0.01	1.73 ± 0.16	<0.001
Hydroxycotinine (ng/mL), mean ± 95%CI	0.20 ± 0.02	0.20 ± 0.03	0.02 ± 0.00	0.03 ± 0.00	0.07 ± 0.00	0.72 ± 0.09	<0.001
Hearing difficulty	6.54	6.42	10.61	4.79	4.69	4.92	0.005
Vision impairment	6.50	5.03	4.58	4.54	6.14	4.94	0.701
Attention Deficit Hyperactivity Disorder	10.37	9.85	8.71	7.87	11.75	11.30	0.228
Walking difficulty	13.36	11.20	10.51	10.19	11.82	12.60	0.721
Difficulty dressing or bathing independently	4.93	3.70	4.37	3.27	3.96	3.11	0.798
Difficulty running errands alone	7.56	6.01	5.64	3.72	6.76	8.01	0.081
**Gender**							0.119
Male	36.68	37.85	34.01	36.37	37.83	43.83	
Female	63.32	62.15	65.99	63.63	62.17	56.17	
**Race/Ethnicity**							<0.001
Non-Hispanic White	31.93	57.22	66.98	58.04	51.99	50.24	
Non-Hispanic African American	29.95	17.90	7.97	14.67	21.43	29.25	
Other	38.12	24.88	25.05	27.29	26.58	20.52	
**Income to poverty ratio^a^**							<0.001
<1.3	34.19	26.00	17.78	21.63	30.08	35.94	
1.3–3.5	34.10	34.46	32.47	34.87	35.71	35.11	
>3.5	21.75	30.70	41.75	31.97	27.30	19.96	
**BMI** <30 kg/m^2^	57.51	58.18	63.58	58.08	53.85	56.32	0.111
**Current drinker^a^**	30.65	36.91	31.01	33.69	41.34	42.62	0.057
**Married/living with partner**	47.97	46.36	37.37	40.60	49.08	59.95	<0.001

* Unweighted data.

a Variables with missing date as another category, the cumulation percent was not 100%.

b Calculated by weighted linear regression model for continuous variables; calculated by weighted chi-squared test for categorical variables. BMI: body mass index.

### Logistic regression analyses

A weighted multivariable logistic regression analysis (Table 2) was conducted to evaluate the relationship between serum cotinine levels (SCL) and its quartiles (Q1–Q4) with health outcomes. In Model 3, participants in the highest quartile of SCL (Q4) exhibited significantly greater odds of walking difficulty compared to those in the lowest quartile (Q4 vs Q1, AOR=2.03; 95% CI: 1.24–3.31, p-trend=0.010).

**Table 2:** Association between serum cotinine level and adverse health outcomes (weighted)

*Variables*	*Model 1*		*Model 2*		*Model 3*	
*Serum cotinine level*	*OR (95% CI)*	*p*	*AOR (95% CI)*	*p*	*AOR (95% CI)*	*p*
Hearing difficulty	0.783 (0.629– 0.976)	**0.035**	0.920 (0.771–1.097)	0.358	0.924 (0.769– 1.109)	0.404
Vision impairment	0.944 (0.824–1.081)	0.408	1.011 (0.890–1.150)	0.864	0.935 (0.794–1.100)	0.423
Attention deficit hyperactivity disorder	0.963 (0.875–1.060)	0.448	1.002 (0.912–1.101)	0.966	0.952 (0.864–1.049)	0.330
Walking difficulty	0.966 (0.882–1.058)	0.465	1.139 (1.038–1.251)	**0.008**	1.117 (0.996–1.253)	0.071
Difficulty dressing or bathing independently	0.927 (0.795–1.079)	0.333	1.050 (0.900–1.225)	0.536	1.023 (0.866–1.208)	0.791
Difficulty running errands alone	0.991 (0.904–1.086)	0.844	1.093 (0.972–1.228)	0.144	1.030 (0.921–1.152)	0.608
**Serum cotinine level** (Quartile)						
**Hearing difficulty**						
Q1 ®	1		1		1	
Q2	0.424 (0.213–0.845)	**0.019**	0.513 (0.241–1.092)	0.091	0.489 (0.224–1.069)	0.086
Q3	0.414 (0.199–0.863)	**0.023**	0.527 (0.238–1.168)	0.122	0.510 (0.226–1.149)	0.118
Q4	0.435 (0.223–0.851)	**0.019**	0.763 (0.365–1.597)	0.478	0.718 (0.370–1.393)	0.337
p for trend	0.734 (0.564–0.954)	**0.025**	0.868 (0.657–1.147)	0.326	0.851 (0.654–1.107)	0.239
**Vision impairment**						
Q1 ®	1		1		1	
Q2	0.992 (0.468–2.104)	0.983	1.115 (0.510–2.437)	0.786	1.096 (0.515–2.334)	0.813
Q3	1.365 (0.673–2.768)	0.394	1.612 (0.779–3.338)	0.206	1.456 (0.729–2.909)	0.298
Q4	1.084 (0.559–2.100)	0.814	1.464 (0.732–2.925)	0.288	0.974 (0.474–2.002)	0.944
p for trend	1.059 (0.872–1.286)	0.568	1.165 (0.950–1.430)	0.150	1.025 (0.837–1.256)	0.813
**Attention deficit hyperactivity disorder**						
Q1 ®	1		1		1	
Q2	0.895 (0.543–1.476)	0.667	0.949 (0.576–1.564)	0.837	0.944 (0.587–1.518)	0.815
Q3	1.395 (0.810–2.403)	0.237	1.526 (0.871–2.676)	0.148	1.434 (0.828–2.484)	0.212
Q4	1.335 (0.879–2.026)	0.182	1.584 (1.034–2.426)	**0.041**	1.228 (0.781–1.930)	0.383
p for trend	1.137 (0.996–1.299)	0.064	1.202 (1.046–1.381)	**0.013**	1.106 (0.956–1.280)	0.188
**Walking difficulty**						
Q1 ®	1		1		1	
Q2	0.966 (0.610–1.532)	0.885	1.245 (0.736–2.107)	0.419	1.372 (0.825–2.284)	0.235
Q3	1.141 (0.691–1.885)	0.609	1.639 (0.949–2.829)	0.084	1.591 (0.877–2.884)	0.140
Q4	1.227 (0.759–1.984)	0.408	2.403 (1.521–3.795)	**<0.001**	2.029 (1.245–3.308)	**0.009**
p for trend	1.080 (0.927–1.259)	0.328	1.335 (1.153–1.546)	**<0.001**	1.256 (1.069–1.475)	**0.010**
**Difficulty dressing or bathing independently**						
Q1 ®	1		1		1	
Q2	0.740 (0.321–1.703)	0.482	0.899 (0.385–2.100)	0.807	0.838 (0.347–2.019)	0.696
Q3	0.902 (0.362–2.247)	0.825	1.169 (0.478–2.863)	0.734	1.058 (0.412–2.719)	0.907
Q4	0.703 (0.398–1.244)	0.234	1.128 (0.666–1.911)	0.657	0.929 (0.510–1.690)	0.811
p for trend	0.914 (0.749–1.116)	0.384	1.061 (0.882–1.276)	0.534	0.998 (0.809–1.232)	0.987
**Difficulty running errands alone**						
Q1 ®	1		1		1	
Q2	0.646 (0.396–1.055)	0.088	0.750 (0.454–1.240)	0.269	0.686 (0.393–1.200)	0.200
Q3	1.211 (0.623–2.355)	0.575	1.515 (0.790–2.904)	0.218	1.269 (0.615–2.621)	0.525
Q4	1.457 (0.919–2.310)	0.117	2.212 (1.431–3.422)	**<0.001**	1.612 (0.936–2.776)	0.099
p for trend	1.187 (1.017–1.387)	**0.036**	1.357 (1.175–1.567)	**<0.001**	1.219 (1.017–1.462)	**0.043**

AOR: adjusted odds ratio. Model 1: no covariates were adjusted. Model 2: age and gender were adjusted. Model 3: age, gender, body mass index, race/ethnicity, data release cycle, education level, marital status, income to poverty ratio, alcohol use, hypertension, diabetes, cancer, and cardiovascular diseases were adjusted. ® Reference categories.

**Figure 3:**
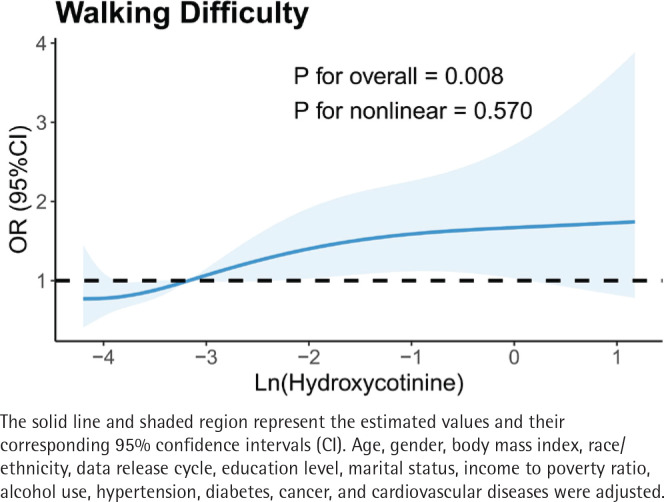
Restricted cubic spline (RCS) model for the relationship between ln-transformation hydroxycotinine and walking difficulty after adjusting all variables

Further analysis of cotinine and hydroxycotinine levels, including their respective quartiles (Q1–Q4), was performed using weighted multivariable logistic regression (Supplementary file Table 3). In Model 3, hydroxycotinine was significantly associated with increased odds of walking difficulty (AOR=1.48; 95% CI: 1.06–2.08, p=0.030). Figure 3 illustrates a positive correlation between ln-transformation hydroxycotinine and walking difficulty. Notably, individuals in the highest quartile of hydroxycotinine (Q4) demonstrated higher odds of walking difficulty (Q4 vs Q1, AOR=1.99; 95% CI: 1.24–3.21, p-trend=0.007) and greater difficulty running errands alone (Q4 vs Q1, AOR=2.09; 95% CI: 1.13–3.88, p-trend=0.036).

### Subgroup analyses

Subgroup analyses, adjusted for covariates, revealed significant heterogeneity in the associations between SCL, cotinine, hydroxycotinine, and their quartiles with physical disabilities, stratified by gender and age (Supplementary file Table 4). Among males, the highest quartiles of cotinine and hydroxycotinine were more strongly associated with walking difficulty compared to females (cotinine, AOR=2.92 vs 1.49; hydroxycotinine, AOR=3.23 vs 1.78; interaction p=0.027 and 0.033, respectively).

**Figure 4:**
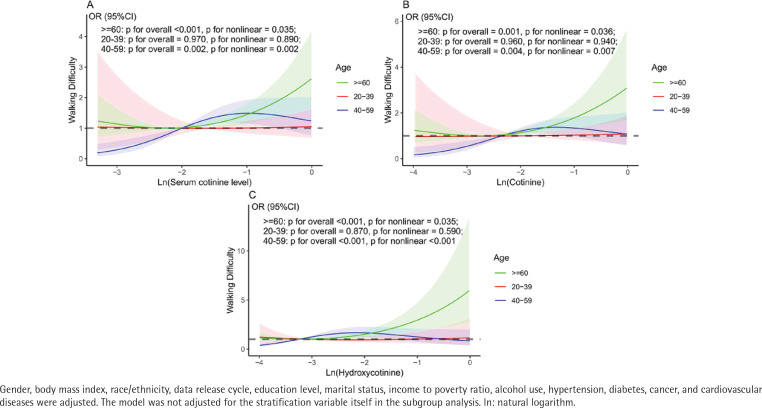
RCS curves for the association between different SHS metabolites and walking difficulty

Stratification by age demonstrated that, in adults aged ≥60 years, the associations of SCL, cotinine, and hydroxycotinine with walking difficulty were stronger compared to those in younger adults. In Model 3, the adjusted odds ratios for walking difficulty were as follows: SCL (AOR=1.58 vs 1.04 vs 0.95), cotinine (AOR=1.80 vs 1.04 vs 0.92), and hydroxycotinine (AOR=4.57 vs 1.20 vs 0.88), with corresponding interaction p-values of 0.016, 0.046, and 0.025, respectively. Figure 4 presents RCS of the natural log-transformed values of SCL, cotinine, and hydroxycotinine in relation to walking difficulty after adjusting for all covariates. A nonlinear relationship between ln(hydroxycotinine) and walking difficulty was observed in adults aged ≥60 years, with an inflection point at -1.306 (Table 3). Beyond this threshold, a statistically significant positive association emerged, indicating that for ln(hydroxycotinine) values greater than -1.306, the odds of experiencing walking difficulty increased notably (AOR=2.57; 95% CI: 1.33–4.96, p=0.005). No significant heterogeneity was observed based on race or ethnicity in the stratified analyses.

**Table 3:** Threshold effect analysis of ln(serum cotinine level), ln(cotinine) and ln(hydroxycotinine) on walking difficulty in older adults (aged≥60 years) using two-piecewise linear regression model

	*AOR (95% CI)*	*p*
**ln (serum cotinine levels)**		
Inflection point	-0.322	
< -0.322 segment effect	1.03 (0.81–1.31)	0.794
> -0.322 segment effect	2.10 (1.17–3.75)	**0.012**
log likelihood ratio	0.057	
**ln (cotinine)**		
Inflection point	-0.596	
< -0.596 segment effect	1.02 (0.83–1.27)	0.819
> -0.596 segment effect	2.02 (1.10–3.74)	**0.024**
log likelihood ratio	0.073	
**ln (hydroxycotinine)**		
Inflection point	-1.306	
< -1.306 segment effect	1.08 (0.84–1.38)	0.551
> -1.306 segment effect	2.57 (1.33–4.96)	**0.005**
log likelihood ratio	**0.033**	

A p<0.05 indicates that the fitting effect between the piecewise linear regression models and the data is significantly better than that of the one-line linear regression model. AOR: adjusted odds ratio. Fully adjusted model: age, gender, body mass index, race/ethnicity, data release cycle, education level, marital status, income to poverty ratio, alcohol use, hypertension, diabetes, cancer, and cardiovascular diseases, were adjusted.

## DISCUSSION

In a nationally representative sample of US adults, SHS exposure was independently associated with physical disabilities among passive smokers, even after adjusting for demographic factors, lifestyle variables, and chronic conditions such as hypertension, cancer, and diabetes. Hydroxycotinine levels showed a significant positive association with walking difficulty, underscoring a stable, independent effect of SHS on motor function. Moreover, quartile analyses further demonstrated that higher exposure, reflected by the upper quartiles of SCL and hydroxycotinine, was significantly associated with walking difficulty. Notably, individuals in the highest quartile of hydroxycotinine exhibited increased odds of difficulty running errands alone, providing additional evidence that passive smoking is a risk factor for lower limb musculoskeletal impairments. Subgroup analyses revealed significant gender differences, with males displaying stronger associations between the highest quartiles of cotinine and hydroxycotinine and walking difficulty compared to females. Age stratification highlighted that adults aged ≥60 years experienced more pronounced associations between serum cotinine, cotinine, hydroxycotinine, and walking difficulty, indicating that older individuals may be particularly vulnerable to the adverse effects of SHS on mobility. Stratified analyses by race/ethnicity revealed no evidence of heterogeneity, indicating that our findings remained consistent across different racial and ethnic groups.

Cotinine, the primary metabolite of nicotine, is commonly used as a biomarker to estimate the daily nicotine intake of individuals exposed to cigarette smoke. Additionally, incorporating hydroxycotinine can enhance the accuracy of nicotine intake estimates. Both cotinine and hydroxycotinine are widely used biomarkers for measuring smoke exposure. Cotinine is considered to have higher specificity, while hydroxycotinine is regarded as a more sensitive biomarker for low-level exposure^15^. Currently, cotinine is more commonly used as a biomarker for SHS exposure. However, since our study focuses on SHS exposure in non-smokers, hydroxycotinine in our research shows a more significant association with various physical disabilities compared to other studies. This finding supports the use of hydroxycotinine and SCL as biomarkers for SHS exposure in non-smokers. Cotinine, on the other hand, may be more suitable for measuring smoke exposure in populations that include smokers, as cotinine concentrations are often above detection limits, making it easier to measure and use^16^.

This study explores the impact of SHS exposure on physical functional impairment in US adults, a topic that has been largely underexplored in prior research, which has predominantly focused on older populations^27-29^. Studies by Akhtar et al.^28^ and García-Esquinas et al.^29^ primarily examined SHS exposure effects on cognitive function and frailty in older adults. While direct comparisons are challenging, our findings are consistent with existing evidence linking SHS exposure to physical disabilities. Notably, our subgroup analysis identifies a significant gap in prior research: adults aged ≥60 years exhibited a stronger association between SHS exposure and walking difficulty compared to younger age groups. In regard to gender differences, our findings diverge from the prevailing literature, which generally reports that men have better physical function than women^30^. In contrast, we observed that men were more adversely affected by SHS exposure in relation to walking difficulty. This suggests that SHS may cause more severe damage to muscle groups in men, possibly related to the lack of estrogen in men and the absence of the important role of estrogen in maintaining muscle mass, a hypothesis that warrants further investigation^31^. Moreover, the higher correlation between SCL and its metabolites and walking difficulty observed in the elderly is in line with the findings of Garcia-Esquinas et al.^29^ that SHS exposure is associated with frailty syndrome in the elderly. We believe that this phenomenon may be related to the atrophy of skeletal muscle in the elderly^32^. Furthermore, our analyses revealed no significant heterogeneity across racial and ethnic groups, suggesting that the relationship between SHS exposure and physical disabilities is consistent across diverse populations. However, the specific role of ethnicity in SHS-related health outcomes remains unclear, underscoring the need for additional research in this area.

The mechanism underlying the association of secondhand smoke with walking difficulty is still uncertain. Most studies have attempted to explain this phenomenon from an immunological perspective. There is substantial evidence demonstrating that SHS is a potent inflammatory stimulus, leading to elevated levels of inflammatory mediators such as C-reactive protein, interleukin-6, and tumor necrosis factor-alpha^33^. Chronic inflammation caused by SHS has been shown to be a risk factor for poor musculoskeletal performance and subsequent decline in physical function among older adults^34^. Experimental evidence also confirms that smoking impairs muscle protein synthesis and increases the expression of genes related to impaired muscle maintenance^35^. A study comparing the skeletal muscle characteristics and fatigue resistance of 45 non-smokers and 40 smokers found that smokers experience greater peripheral muscle fatigue than non-smokers^36^. In summary, current discussions regarding the mechanisms underlying the decline in physical function among adults due to passive smoking primarily attribute it to chronic inflammation induced by SHS, which subsequently leads to poor musculoskeletal physiology^27,29,34^.

### Strengths and limitations

This study benefits from the use of serum cotinine and hydroxycotinine as biomarkers, providing more accurate associations with health outcomes. The NHANES dataset also ensures the generalizability of our findings. However, the cross-sectional nature of the data (2013–2018) limits our ability to capture long-term trends, and biomarkers only reflect recent exposure, potentially underestimating long-term effects. Despite the use of DAG analysis, we are still unable to completely rule out the potential influence of confounding factors on the results. Our analysis suggests that elevated cotinine levels are associated with increased risks of physical decline in US adults. Longitudinal studies are needed to further elucidate these relationships, and efforts to protect non-smokers, particularly those with chronic conditions, from SHS exposure are crucial to prevent functional impairments.

## CONCLUSIONS

This study provides evidence that serum cotinine levels independently contribute to the occurrence of various physical disabilities. This finding enhances our understanding of the harmful effects of cigarette smoke on physical functions, underscoring the importance of smoking cessation for public health. Consequently, our research on passive smoking and functional impairments can provide support for the development and implementation of smoke-free policies. This will not only help raise public awareness of healthy lifestyles but also reduce functional impairment issues caused by smoking, ultimately promoting the overall health and well-being of society.

## Supplementary Material



## Data Availability

The data supporting this research are available from the following source: https://wwwn.cdc.gov/nchs/nhanes/.
