# Supplementary material for: Exposure to secondhand smoke and physical disabilities in non-smokers: A national cross-sectional study with cotinine measurements from NHANES 2013–2018
Source: Tob Induc Dis. 2025 Feb 21;23:10.18332/tid/200546. doi: 10.18332/tid/200546 (PMC11843551; doi:10.18332/tid/200546)
Supplement: Supplementary file 1 [file TID-23-18-s1.pdf]

Supplementary materials

Exposure to secondhand smoke and physical disabilities in non-smokers: a national cross-sectional study with cotinine measurement

Jiahui He[1,2], Zhounan Wu[1,2], Yuhang Liang[1,2], Jinshen He, MD[1, *]

[1] Department of Orthopaedic Surgery, Third Xiangya Hospital of Central South University, Changsha, Hunan, China, 410013

[2] Xiangya School of Medicine, Central South University, Changsha, China, 410013

*** Corresponding author**

Jinshen He, MD

Institutional address: Department of Orthopaedic Surgery, Third Xiangya Hospital of Central South University, Changsha, Hunan 410013, China

Email: Jinshen.he@hotmail.com

Tables legends:

Supplementary Table 1. Some other characteristics of participants, Weighted.

Supplementary Table 2. Variance Inflation Factor (VIF) test for each confounding factors.

Supplementary Table 3. Association between cotinine, hydroxycotinine and adverse health outcomes, Weighted.

Supplementary Table 4-1. Association between serum cotinine level, cotinine, hydroxycotinine and adverse health outcomes, stratified by gender, Weighted.

Supplementary Table 4-2. Association between serum cotinine level, cotinine, hydroxycotinine and adverse health outcomes, stratified by age, Weighted.

Supplementary Table 4-3. Association between serum cotinine level, cotinine, hydroxycotinine and adverse health outcomes, stratified by race/ethnicity, Weighted.

Supplementary Table 1. Some other characteristics of participants, Weighted.

	Total[*]	Total	Quartiles (ng/mL Serum cotinine level)				
			**Q1**	**Q2**	**Q3**	**Q4**	***P***
**Education level**							**<0.001**
Less than high school	20.32%	13.26%	10.43%	12.33%	14.29%	16.49%	
High school	24.74%	25.95%	18.66%	29.23%	24.32%	32.73%	
More than high school	54.93%	60.79%	70.91%	58.44%	61.39%	50.78%	
**Data release cycle**							0.897
2013-2014	35.94%	33.75%	34.84%	32.43%	33.81%	33.78%	
2015-2016	32.76%	32.89%	33.64%	34.55%	29.86%	33.35%	
2017-2018	31.29%	33.36%	31.52%	33.02%	36.34%	32.87%	
**Diabetes**	14.33%	10.24%	12.36%	7.85%	9.57%	10.89%	0.164
**Hypertension**	34.61%	30.43%	34.18%	30.86%	29.63%	26.43%	0.178
**Cancer**	6.36%	7.19%	7.97%	8.92%	5.81%	5.90%	0.366
**Cardiovascular vascular disease**	8.80%	7.26%	10.47%	4.87%	5.66%	7.54%	**0.005**

[*], the unweighted data;

[#], variables with missing date as another category, the cumulation percent was not 100%;

Mean±95%CI for continuous variables, ***P*** value was calculated by weighted linear regression model;

% for categorical variables, ***P*** value was calculated by weighted chi-square test.

Supplementary Table 2. Variance Inflation Factor (VIF) test for each confounding factors.*

Confounding factors	VIF
**Race/Ethnicity**	1.127
**Gender**	1.033
**Age**	1.587
**Education level**	1.106
**Marital status**	1.031
**Income to poverty ratio**	1.020
**Hypertension**	1.407
**Body Mass Index**	1.066
**Diabetes**	1.219
**Cardiovascular Diseases**	1.175

**Cancer**	1.083
**Data Release Cycle**	1.021
**Alcohol use**	1.157

*: The variance expansion coefficient (VIF) is less than 5, indicating that there is no collinearity relationship between confounding factors.

Supplementary Table 3. Association between cotinine, hydroxycotinine and adverse health outcomes, Weighted.

**Variables**		**Model 1**		**Model 2**		**Model 3**	
**Cotinine**		**OR (95% CI)**	***P***	**OR (95% CI)**	***P***	**OR (95% CI)**	***P***
**Hearing difficulty**		**0.640 (0.452, 0.905)**	**0.015**	0.862 (0.660, 1.127)	0.283	0.884 (0.672, 1.162)	0.385
**Vision impairment**		0.917 (0.755, 1.114)	0.388	1.020 (0.856, 1.216)	0.823	0.915 (0.731, 1.145)	0.446
**Attention deficit hyperactivity disorder**		0.942 (0.829, 1.071)	0.370	0.997 (0.884, 1.124)	0.961	0.934 (0.821, 1.063)	0.310
**Walking difficulty**		0.913 (0.798, 1.044)	0.189	**1.167 (1.029, 1.325)**	**0.021**	1.135 (0.972, 1.325)	0.121
**Difficulty dressing or bathing independently**		0.878 (0.710, 1.088)	0.241	1.072 (0.880, 1.306)	0.491	1.031 (0.836, 1.272)	0.776
**Difficulty running errands alone**		0.955 (0.844, 1.081)	0.473	1.106 (0.956, 1.278)	0.182	1.018 (0.886, 1.170)	0.802
**Cotinine(Quartile)**							
**Hearing difficulty**	**Q1**	Reference		Reference		Reference	
	**Q2**	**0.269 (0.154, 0.472**	**<0.001**	**0.340 (0.174, 0.664)**	**0.003**	**0.297 (0.146, 0.604)**	**0.003**
	**Q3**	**0.325 (0.149, 0.708)**	**0.007**	0.447 (0.194, 1.029)	0.066	0.459 (0.190, 1.111)	0.098
	**Q4**	**0.331 (0.189, 0.581)**	**<0.001**	0.685 (0.362, 1.296)	0.251	0.617 (0.355, 1.072)	0.100
	***P* for trend**	**0.001**		0.121		0.084	
**Vision impairment**	**Q1**	Reference		Reference		Reference	
	**Q2**	0.797 (0.400, 1.588)	0.523	0.947 (0.464, 1.933)	0.882	0.877 (0.448, 1.715)	0.705
	**Q3**	1.333 (0.652, 2.725)	0.436	1.665 (0.790, 3.507)	0.188	1.527 (0.736, 3.170)	0.267
	**Q4**	0.915 (0.482, 1.738)	0.787	1.343 (0.685, 2.635)	0.396	0.857 (0.417, 1.760)	0.678
	***P* for trend**	0.795		0.169		0.883	
**Attention deficit hyperactivity disorder**	**Q1**	Reference		Reference		Reference	
	**Q2**	0.990 (0.587, 1.672)	0.972	1.098 (0.634, 1.902)	0.741	1.094 (0.620, 1.931)	0.758
	**Q3**	1.326 (0.742, 2.369)	0.346	1.513 (0.822, 2.785)	0.191	1.478 (0.779, 2.803)	0.244
	**Q4**	1.352 (0.830, 2.202)	0.232	1.699 (0.998, 2.893)	0.058	1.307 (0.720, 2.373)	0.388

	***P* for trend**	0.129		**0.027**		0.244	
**Walking difficulty**	**Q1**	Reference		Reference		Reference	
	**Q2**	0.741 (0.464, 1.184)	0.217	1.053 (0.599, 1.849)	0.859	1.060 (0.570, 1.974)	0.855
	**Q3**	1.028 (0.580, 1.823)	0.924	1.651 (0.910, 2.993)	0.107	1.670 (0.883, 3.157)	0.128
	**Q4**	0.928 (0.587, 1.468)	0.752	**2.136 (1.394, 3.274)**	**0.001**	1.681 (0.984, 2.872)	0.070
	***P* for trend**	0.960		**0.001**		**0.036**	
**Difficulty dressing or bathing independently**	**Q1**	Reference		Reference		Reference	
	**Q2**	0.818 (0.330, 2.026)	0.666	1.095 (0.439, 2.729)	0.847	0.973 (0.376, 2.516)	0.955
	**Q3**	0.897 (0.354, 2.274)	0.820	1.277 (0.529, 3.083)	0.589	1.203 (0.487, 2.968)	0.693
	**Q4**	0.724 (0.381, 1.375)	0.329	1.367 (0.788, 2.371)	0.273	1.066 (0.585, 1.943)	0.836
	***P* for trend**	0.413		0.220		0.667	
**Difficulty running errands alone**	**Q1**	Reference		Reference		Reference	
	**Q2**	0.798 (0.459, 1.385)	0.426	1.014 (0.573, 1.795)	0.961	0.902 (0.493, 1.649)	0.740
	**Q3**	1.164 (0.612, 2.214)	0.647	1.580 (0.845, 2.953)	0.160	1.406 (0.682, 2.898)	0.366
	**Q4**	1.404 (0.882, 2.234)	0.160	**2.434 (1.577, 3.758)**	**<0.001**	1.702 (1.010, 2.867)	0.058
	***P* for trend**	0.083		**<0.001**		**0.030**	
**Hydroxycotinine**							
**Hearing difficulty**		0.701 (0.380, 1.292)	0.261	0.882 (0.555, 1.401)	0.597	0.841 (0.515, 1.372)	0.494
**Vision impairment**		0.879 (0.596, 1.298)	0.521	1.009 (0.689, 1.477)	0.963	0.810 (0.485, 1.353)	0.428
**Attention deficit hyperactivity disorder**		0.935 (0.676, 1.295)	0.689	1.034 (0.739, 1.445)	0.847	0.875 (0.608, 1.260)	0.479
**Walking difficulty**		1.087 (0.843, 1.401)	0.524	**1.550 (1.189, 2.021)**	**0.003**	**1.485 (1.059, 2.081)**	**0.030**
**Difficulty dressing or bathing independently**		0.900 (0.575, 1.406)	0.645	1.126 (0.703, 1.803)	0.625	1.063 (0.619, 1.826)	0.826
**Difficulty running errands alone**		1.117 (0.821, 1.520)	0.485	1.372 (0.925, 2.035)	0.123	1.191 (0.781, 1.816)	0.426
**Hydroxycotinine (Quartile)**							
**Hearing difficulty**	**Q1**	Reference		Reference		Reference	
	**Q2**	1.136 (0.647, 1.994)	0.660	0.982 (0.525, 1.836)	0.954	1.064 (0.574, 1.972)	0.846
	**Q3**	0.741 (0.448, 1.226)	0.250	0.689 (0.386, 1.229)	0.214	0.666 (0.370, 1.197)	0.187
	**Q4**	0.817 (0.451, 1.478)	0.507	0.976 (0.520, 1.829)	0.939	0.971 (0.519, 1.817)	0.927
	***P* for trend**	0.284		0.637		0.542	
**Vision impairment**	**Q1**	Reference		Reference		Reference	
	**Q2**	1.025 (0.541, 1.941)	0.941	0.964 (0.519, 1.791)	0.908	0.990 (0.517, 1.896)	0.977

	**Q3**	1.262 (0.706, 2.256)	0.437	1.300 (0.708, 2.388)	0.403	1.224 (0.684, 2.189)	0.503
	**Q4**	1.148 (0.637, 2.068)	0.648	1.288 (0.689, 2.409)	0.433	0.907 (0.452, 1.821)	0.786
	***P* for trend**	0.528		0.314		0.952	
**Attention deficit hyperactivity disorder**	**Q1**	Reference		Reference		Reference	
	**Q2**	1.321 (0.789, 2.212)	0.296	1.283 (0.765, 2.151)	0.350	1.259 (0.706, 2.245)	0.444
	**Q3**	1.626 (0.913, 2.898)	0.106	1.662 (0.935, 2.955)	0.091	1.455 (0.811, 2.610)	0.221
	**Q4**	1.492 (0.953, 2.336)	0.088	**1.599 (1.037, 2.467)**	**0.04**	1.199 (0.739, 1.944)	0.469
	***P* for trend**	0.055		**0.021**		0.379	
**Walking difficulty**	**Q1**	Reference		Reference		Reference	
	**Q2**	1.295 (0.765, 2.193)	0.342	1.162 (0.716, 1.888)	0.547	1.249 (0.797, 1.958)	0.342
	**Q3**	1.538 (0.932, 2.537)	0.099	1.687 (1.013, 2.809)	0.051	1.634 (0.969, 2.755)	0.078
	**Q4**	**1.699 (1.028, 2.807)**	**0.045**	**2.250 (1.417, 3.572)**	**0.001**	**1.991 (1.237, 3.206)**	**0.009**
	***P* for trend**	**0.026**		**<0.001**		**0.007**	
**Difficulty dressing or bathing independently**	**Q1**	Reference		Reference		Reference	
	**Q2**	1.263 (0.623, 2.559)	0.521	1.140 (0.542, 2.399)	0.731	1.067 (0.462, 2.466)	0.881
	**Q3**	1.312 (0.564, 3.052)	0.532	1.348 (0.576, 3.155)	0.496	1.194 (0.480, 2.971)	0.707
	**Q4**	0.904 (0.506, 1.614)	0.735	1.051 (0.573, 1.931)	0.872	0.873 (0.417, 1.831)	0.723
	***P* for trend**	0.839		0.696		0.867	
**Difficulty running errands alone**	**Q1**	Reference		Reference		Reference	
	**Q2**	1.786 (1.033, 3.086)	0.044	1.665 (0.962, 2.885)	0.076	1.628 (0.937, 2.828)	0.097
	**Q3**	1.825 (0.918, 3.626)	0.093	1.909 (0.957, 3.809)	0.074	1.609 (0.763, 3.395)	0.224
	**Q4**	**2.347 (1.349, 4.083)**	**0.004**	**2.765 (1.600, 4.778)**	**<0.001**	**2.092 (1.128, 3.880)**	**0.028**
	***P* for trend**	**0.007**		**<0.001**		**0.036**	

Model 1: no covariates were adjusted.

Model 2: age and gender were adjusted.

Model 3: age, gender, body mass index, race/ethnicity, data release cycle, education level, marital status, income to poverty ratio, alcohol use, hypertension, diabetes, cancer, and cardiovascular diseases were adjusted.

Supplementary Table 4-1. Association between serum cotinine level, cotinine, hydroxycotinine and adverse health outcomes, stratified by gender, Weighted.

Model 1	Model 2	Model 3

	Men (n=796)	Women (n=1,374)	*P*-interaction	Men (n=796)	Women (n=1,374)	*P*-interaction	Men (n=796)	Women (n=1,374)	*P*-interaction
**Hearing difficulty**	**OR (95%CI) *P*-value**	**OR (95%CI) *P*-value**		**OR (95%CI) *P*-value**	**OR (95%CI) *P*-value**		**OR (95%CI) *P*-value**	**OR (95%CI) *P*-value**	
**Serum cotinine level**	0.833 (0.663, 1.047) 0.124	0.643 (0.393, 1.053) 0.087	0.354	0.960 (0.793, 1.163) 0.680	0.818 (0.524, 1.275) 0.380	0.517	0.969 (0.792, 1.186) 0.763	0.813 (0.512, 1.290) 0.388	0.497
**Vision impairment**	**OR (95%CI) *P*-value**	**OR (95%CI) *P*-value**		**OR (95%CI) *P*-value**	**OR (95%CI) *P*-value**		**OR (95%CI) *P*-value**	**OR (95%CI) *P*-value**	
**Serum cotinine level**	0.952 (0.766, 1.182) 0.657	0.948 (0.783, 1.147) 0.584	0.977	0.999 (0.811, 1.231) 0.996	1.026 (0.864, 1.218) 0.769	0.857	0.946 (0.754, 1.186) 0.632	0.922 (0.716, 1.186) 0.531	0.886
**Attention deficit hyperactivity disorder**	**OR (95%CI) *P*-value**	**OR (95%CI) *P*-value**		**OR (95%CI) *P*-value**	**OR (95%CI) *P*-value**		**OR (95%CI) *P*-value**	**OR (95%CI) *P*-value**	
**Serum cotinine level**	0.925 (0.791, 1.081) 0.332	1.023 (0.905, 1.155) 0.722	0.321	0.942 (0.803, 1.105) 0.469	1.053 (0.942, 1.176) 0.372	0.261	0.895 (0.742, 1.078) 0.254	0.997 (0.873, 1.140) 0.970	0.393
**Walking difficulty**	**OR (95%CI) *P*-value**	**OR (95%CI) *P*-value**		**OR (95%CI) *P*-value**	**OR (95%CI) *P*-value**		**OR (95%CI) *P*-value**	**OR (95%CI) *P*-value**	
**Serum cotinine level**	0.912 (0.786, 1.060) 0.238	1.041 (0.917, 1.181) 0.540	0.224	1.016 (0.878, 1.176) 0.830	**1.241 (1.105, 1.395) 0.001**	**0.041**	1.036 (0.898, 1.196) 0.631	1.176 (1.005, 1.378) 0.055	0.226
**Difficulty dressing or bathing independently**	**OR (95%CI) *P*-value**	**OR (95%CI) *P*-value**		**OR (95%CI) *P*-value**	**OR (95%CI) *P*-value**		**OR (95%CI) *P*-value**	**OR (95%CI) *P*-value**	
**Serum cotinine level**	0.958 (0.780, 1.177) 0.685	0.897 (0.713, 1.127) 0.355	0.668	1.054 (0.853, 1.303) 0.629	1.045 (0.860, 1.270) 0.662	0.950	1.057 (0.847, 1.319) 0.626	0.979 (0.791, 1.211) 0.844	0.596
**Difficulty running errands alone**	**OR (95%CI) *P*-value**	**OR (95%CI) *P*-value**		**OR (95%CI) *P*-value**	**OR (95%CI) *P*-value**		**OR (95%CI) *P*-value**	**OR (95%CI) *P*-value**	

**Serum cotinine level**	0.979 (0.832, 1.152) 0.799	1.030 (0.908, 1.168) 0.649	0.633	1.045 (0.871, 1.252) 0.640	**1.140 (1.010, 1.288) 0.041**	0.401	1.007 (0.814, 1.246) 0.947	1.051 (0.916, 1.207) 0.483	0.762
**Hearing difficulty**	**OR (95%CI) *P*-value**	**OR (95%CI) *P*-value**		**OR (95%CI) *P*-value**	**OR (95%CI) *P*-value**		**OR (95%CI) *P*-value**	**OR (95%CI) *P*-value**	
**Serum cotinine level (Quartile)**			0.877			0.878			0.714
**Q1**	Reference	Reference		Reference	Reference		Reference	Reference	
**Q2**	0.368 (0.117, 1.157) 0.095	**0.461 (0.218, 0.972) 0.049**		0.458 (0.138, 1.512) 0.208	0.559 (0.244, 1.282) 0.178		0.422 (0.128, 1.388) 0.171	0.539 (0.219, 1.331) 0.195	
**Q3**	0.462 (0.145, 1.475) 0.200	**0.360 (0.140, 0.926) 0.040**		0.630 (0.181, 2.188) 0.472	0.451 (0.158, 1.292) 0.147		0.685 (0.204, 2.300) 0.547	0.394 (0.139, 1.118) 0.095	
**Q4**	0.388 (0.145, 1.037) 0.067	0.452 (0.203, 1.007) 0.059		0.854 (0.308, 2.367) 0.763	0.698 (0.277, 1.761) 0.451		0.865 (0.329, 2.272) 0.771	0.612 (0.249, 1.503) 0.297	
**Vision impairment**	**OR (95%CI) *P*-value**	**OR (95%CI) *P*-value**		**OR (95%CI) *P*-value**	**OR (95%CI) *P*-value**		**OR (95%CI) *P*-value**	**OR (95%CI) *P*-value**	
**Serum cotinine level (Quartile)**			**0.020**			**0.018**			**0.004**
**Q1**	Reference	Reference		Reference	Reference		Reference	Reference	
**Q2**	0.512 (0.148, 1.774) 0.298	1.587 (0.708, 3.559) 0.269		0.566 (0.156, 2.055) 0.393	1.787 (0.766, 4.166) 0.187		0.405 (0.120, 1.364) 0.160	2.127 (0.992, 4.563) 0.069	
**Q3**	0.350 (0.110, 1.112) 0.083	**2.759 (1.250, 6.089) 0.016**		0.401 (0.127, 1.267) 0.128	**3.283 (1.461, 7.379) 0.007**		0.336 (0.115, 0.980) 0.060	**3.150 (1.456, 6.814) 0.009**	
**Q4**	0.504 (0.174, 1.458) 0.214	1.891 (0.845, 4.235) 0.130		0.698 (0.238, 2.049) 0.517	**2.440 (1.069, 5.570) 0.041**		0.432 (0.154, 1.213) 0.127	1.685 (0.700, 4.059) 0.258	
**Attention deficit hyperactivity disorder**	**OR (95%CI) *P*-value**	**OR (95%CI) *P*-value**		**OR (95%CI) *P*-value**	**OR (95%CI) *P*-value**		**OR (95%CI) *P*-value**	**OR (95%CI) *P*-value**	
**Serum cotinine level (Quartile)**			0.147			0.169			0.190

**Q1**	Reference	Reference		Reference	Reference		Reference	Reference	
**Q2**	1.347 (0.582, 3.118) 0.490	0.740 (0.382, 1.433) 0.378		1.407 (0.590, 3.355) 0.446	0.776 (0.402, 1.500) 0.456		1.208 (0.494, 2.958) 0.683	0.811 (0.438, 1.500) 0.512	
**Q3**	1.394 (0.486, 3.998) 0.540	1.431 (0.774, 2.647) 0.260		1.482 (0.503, 4.369) 0.480	1.541 (0.818, 2.902) 0.189		1.407 (0.464, 4.266) 0.553	1.439 (0.782, 2.650) 0.256	
**Q4**	0.971 (0.416, 2.270) 0.947	1.638 (0.933, 2.874) 0.094		1.117 (0.448, 2.785) 0.813	**1.837 (1.056, 3.194) 0.038**		0.704 (0.267, 1.859) 0.487	1.578 (0.862, 2.889) 0.154	
**Walking difficulty**	**OR (95%CI) *P*-value**	**OR (95%CI) *P*-value**		**OR (95%CI) *P*-value**	**OR (95%CI) *P*-value**		**OR (95%CI) *P*-value**	**OR (95%CI) *P*-value**	
**Serum cotinine level (Quartile)**			**0.042**			0.079			0.076
**Q1**	Reference	Reference		Reference	Reference		Reference	Reference	
**Q2**	2.044 (0.872, 4.795) 0.108	0.782 (0.478, 1.277) 0.332		**2.776 (1.049, 7.347) 0.047**	0.961 (0.550, 1.679) 0.889		**2.952 (1.117, 7.804) 0.041**	1.071 (0.609, 1.882) 0.815	
**Q3**	**2.526 (1.111, 5.744) 0.033**	0.906 (0.496, 1.653) 0.749		**3.752 (1.504, 9.358) 0.007**	1.227 (0.634, 2.375) 0.547		**4.360 (1.701, 11.176) 0.006**	1.091 (0.543, 2.194) 0.809	
**Q4**	1.316 (0.633, 2.734) 0.466	1.380 (0.769, 2.476) 0.287		**2.708 (1.283, 5.713) 0.013**	**2.452 (1.397, 4.304) 0.004**		**2.811 (1.384, 5.711) 0.010**	1.901 (1.029, 3.513) 0.054	
**Difficulty dressing or bathing independently**	**OR (95%CI) *P*-value**	**OR (95%CI) *P*-value**		**OR (95%CI) *P*-value**	**OR (95%CI) *P*-value**		**OR (95%CI) *P*-value**	**OR (95%CI) *P*-value**	
**Serum cotinine level (Quartile)**			0.620			0.678			0.801
**Q1**	Reference	Reference		Reference	Reference		Reference	Reference	
**Q2**	1.478 (0.365, 5.986) 0.587	0.525 (0.190, 1.450) 0.222		1.858 (0.410, 8.407) 0.427	0.625 (0.225, 1.742) 0.375		1.509 (0.316, 7.200) 0.612	0.626 (0.203, 1.932) 0.425	
**Q3**	1.512 (0.288, 7.947) 0.628	0.726 (0.308, 1.708) 0.467		2.005 (0.341, 11.792) 0.446	0.925 (0.393, 2.176) 0.858		1.908 (0.294, 12.362) 0.506	0.808 (0.338, 1.932) 0.637	

**Q4**	0.844 (0.244, 2.916) 0.790	0.700 (0.350, 1.403) 0.322		1.527 (0.402, 5.799) 0.538	1.027 (0.550, 1.916) 0.935		1.331 (0.278, 6.379) 0.724	0.817 (0.399, 1.672) 0.586	
**Difficulty running errands alone**	**OR (95%CI) *P*-value**	**OR (95%CI) *P*-value**		**OR (95%CI) *P*-value**	**OR (95%CI) *P*-value**		**OR (95%CI) *P*-value**	**OR (95%CI) *P*-value**	
**Serum cotinine level (Quartile)**			0.948			0.938			0.993
**Q1**	Reference	Reference		Reference	Reference		Reference	Reference	
**Q2**	0.870 (0.289, 2.625) 0.807	0.586 (0.311, 1.105) 0.107		1.013 (0.304, 3.371) 0.984	0.672 (0.354, 1.277) 0.233		0.802 (0.237, 2.717) 0.727	0.649 (0.323, 1.306) 0.240	
**Q3**	1.395 (0.306, 6.352) 0.670	1.185 (0.614, 2.287) 0.615		1.713 (0.347, 8.468) 0.513	1.464 (0.769, 2.789) 0.254		1.441 (0.277, 7.502) 0.669	1.215 (0.617, 2.394) 0.579	
**Q4**	1.815 (0.563, 5.848) 0.324	1.425 (0.817, 2.484) 0.220		2.880 (0.853, 9.717) 0.097	**1.995 (1.194, 3.332) 0.012**		1.903 (0.478, 7.578) 0.372	1.510 (0.806, 2.831) 0.213	
**Hearing difficulty**	**OR (95%CI) *P*-value**	**OR (95%CI) *P*-value**		**OR (95%CI) *P*-value**	**OR (95%CI) *P*-value**		**OR (95%CI) *P*-value**	**OR (95%CI) *P*-value**	
**Cotinine**	0.713 (0.501, 1.016) 0.068	0.468 (0.203, 1.078) 0.082	0.370	0.914 (0.686, 1.218) 0.542	0.743 (0.386, 1.432) 0.380	0.569	0.947 (0.698, 1.285) 0.731	0.747 (0.388, 1.440) 0.392	0.519
**Vision impairment**	**OR (95%CI) *P*-value**	**OR (95%CI) *P*-value**		**OR (95%CI) *P*-value**	**OR (95%CI) *P*-value**		**OR (95%CI) *P*-value**	**OR (95%CI) *P*-value**	
**Cotinine**	0.934 (0.685, 1.275) 0.671	0.915 (0.696, 1.202) 0.526	0.924	1.008 (0.752, 1.350) 0.959	1.035 (0.820, 1.306) 0.776	0.898	0.933 (0.682, 1.277) 0.668	0.894 (0.629, 1.271) 0.538	0.865
**Attention deficit hyperactivity disorder**	**OR (95%CI) *P*-value**	**OR (95%CI) *P*-value**		**OR (95%CI) *P*-value**	**OR (95%CI) *P*-value**		**OR (95%CI) *P*-value**	**OR (95%CI) *P*-value**	
**Cotinine**	0.888 (0.713, 1.106) 0.294	1.016 (0.857, 1.204) 0.857	0.371	0.914 (0.730, 1.143) 0.434	1.061 (0.915, 1.230) 0.441	0.300	0.857 (0.651, 1.129) 0.284	0.989 (0.825, 1.184) 0.903	0.449
**Walking difficulty**	**OR (95%CI) *P*-value**	**OR (95%CI) *P*-value**		**OR (95%CI) *P*-value**	**OR (95%CI) *P*-value**		**OR (95%CI) *P*-value**	**OR (95%CI) *P*-value**	

**Cotinine**	0.827 (0.672, 1.018) 0.080	1.004 (0.841, 1.198) 0.966	0.186	0.986 (0.812, 1.196) 0.884	**1.302 (1.112, 1.524) 0.002**	**0.030**	1.026 (0.850, 1.238) 0.792	1.207 (0.981, 1.485) 0.088	0.229
**Difficulty dressing or bathing independently**	**OR (95%CI) *P*-value**	**OR (95%CI) *P*-value**		**OR (95%CI) *P*-value**	**OR (95%CI) *P*-value**		**OR (95%CI) *P*-value**	**OR (95%CI) *P*-value**	
**Cotinine**	0.939 (0.716, 1.230) 0.648	0.818 (0.593, 1.128) 0.228	0.507	1.090 (0.837, 1.421) 0.526	1.048 (0.816, 1.345) 0.717	0.817	1.099 (0.850, 1.420) 0.479	0.946 (0.703, 1.273) 0.717	0.414
**Difficulty running errands alone**	**OR (95%CI) *P*-value**	**OR (95%CI) *P*-value**		**OR (95%CI) *P*-value**	**OR (95%CI) *P*-value**		**OR (95%CI) *P*-value**	**OR (95%CI) *P*-value**	
**Cotinine**	0.938 (0.771, 1.141) 0.524	1.001 (0.836, 1.198) 0.991	0.638	1.038 (0.839, 1.283) 0.736	1.167 (0.983, 1.384) 0.085	0.372	0.988 (0.772, 1.263) 0.924	1.044 (0.858, 1.271) 0.672	0.753
**Hearing difficulty**	**OR (95%CI) *P*-value**	**OR (95%CI) *P*-value**		**OR (95%CI) *P*-value**	**OR (95%CI) *P*-value**		**OR (95%CI) *P*-value**	**OR (95%CI) *P*-value**	
**Cotinine (Quartile)**			0.946			0.956			0.715
**Q1**	Reference	Reference		Reference	Reference		Reference	Reference	
**Q2**	**0.232 (0.099, 0.546) 0.002**	**0.284 (0.125, 0.644) 0.005**		**0.306 (0.117, 0.798) 0.020**	**0.372 (0.155, 0.892) 0.033**		**0.246 (0.091, 0.664) 0.012**	**0.350 (0.143, 0.859) 0.033**	
**Q3**	0.306 (0.097, 0.966) 0.051	**0.313 (0.135, 0.724) 0.010**		0.474 (0.138, 1.636) 0.245	0.423 (0.173, 1.036) 0.068		0.540 (0.163, 1.786) 0.324	0.401 (0.153, 1.050) 0.078	
**Q4**	**0.267 (0.112, 0.634) 0.005**	**0.358 (0.168, 0.764) 0.012**		0.682 (0.271, 1.716) 0.421	0.686 (0.293, 1.604) 0.390		0.656 (0.270, 1.594) 0.363	0.582 (0.258, 1.311) 0.206	
**Vision impairment**	**OR (95%CI) *P*-value**	**OR (95%CI) *P*-value**		**OR (95%CI) *P*-value**	**OR (95%CI) *P*-value**		**OR (95%CI) *P*-value**	**OR (95%CI) *P*-value**	
**Cotinine (Quartile)**			**0.023**			0.023			**0.003**
**Q1**	Reference	Reference		Reference	Reference		Reference	Reference	
**Q2**	0.330 (0.097, 1.120) 0.083	1.338 (0.623, 2.872) 0.460		0.387 (0.113, 1.324) 0.139	1.571 (0.716, 3.450) 0.267		0.275 (0.081, 0.936) 0.052	1.706 (0.835, 3.486) 0.158	

**Q3**	0.430 (0.134, 1.377) 0.163	**2.458 (1.172, 5.155) 0.022**		0.536 (0.167, 1.726) 0.303	**3.010 (1.410, 6.428) 0.007**		0.421 (0.140, 1.269) 0.140	**3.065 (1.454, 6.462) 0.008**	
**Q4**	0.428 (0.143, 1.279) 0.137	1.522 (0.684, 3.384) 0.310		0.640 (0.213, 1.925) 0.432	2.115 (0.941, 4.755) 0.078		0.380 (0.124, 1.161) 0.105	1.372 (0.560, 3.365) 0.497	
**Attention deficit hyperactivity disorder**	**OR (95%CI) *P*-value**	**OR (95%CI) *P*-value**		**OR (95%CI) *P*-value**	**OR (95%CI) *P*-value**		**OR (95%CI) *P*-value**	**OR (95%CI) *P*-value**	
**Cotinine (Quartile)**			0.194			0.211			0.242
**Q1**	Reference	Reference		Reference	Reference		Reference	Reference	
**Q2**	1.821 (0.685, 4.844) 0.237	0.748 (0.379, 1.478) 0.408		1.973 (0.708, 5.498) 0.202	0.802 (0.400, 1.608) 0.538		1.629 (0.554, 4.789) 0.386	0.848 (0.430, 1.669) 0.638	
**Q3**	1.246 (0.387, 4.009) 0.715	1.439 (0.745, 2.782) 0.285		1.385 (0.416, 4.616) 0.599	1.577 (0.790, 3.148) 0.205		1.188 (0.324, 4.352) 0.798	1.602 (0.802, 3.199) 0.197	
**Q4**	1.180 (0.459, 3.033) 0.733	1.605 (0.938, 2.747) 0.093		1.422 (0.511, 3.961) 0.504	**1.870 (1.070, 3.267) 0.034**		0.884 (0.285, 2.739) 0.833	1.578 (0.830, 3.001) 0.179	
**Walking difficulty**	**OR (95%CI) *P*-value**	**OR (95%CI) *P*-value**		**OR (95%CI) *P*-value**	**OR (95%CI) *P*-value**		**OR (95%CI) *P*-value**	**OR (95%CI) *P*-value**	
**Cotinine (Quartile)**			**0.024**			**0.050**			**0.027**
**Q1**	Reference	Reference		Reference	Reference		Reference	Reference	
**Q2**	2.010 (0.860, 4.701) 0.115	**0.551 (0.316, 0.962) 0.043**		**3.041 (1.099, 8.415) 0.039**	0.720 (0.383, 1.353) 0.314		**3.121 (1.177, 8.275) 0.033**	0.719 (0.349, 1.481) 0.382	
**Q3**	2.018 (0.828, 4.920) 0.131	0.914 (0.479, 1.745) 0.786		**3.462 (1.323, 9.059) 0.016**	1.338 (0.675, 2.651) 0.409		**4.014 (1.518, 10.617) 0.011**	1.273 (0.620, 2.613) 0.519	
**Q4**	1.173 (0.555, 2.480) 0.679	1.038 (0.590, 1.828) 0.897		**2.842 (1.310, 6.163) 0.012**	**2.113 (1.246, 3.583) 0.009**		**2.920 (1.430, 5.965) 0.008**	1.493 (0.757, 2.945) 0.261	
**Difficulty dressing or bathing independently**	**OR (95%CI) *P*-value**	**OR (95%CI) *P*-value**		**OR (95%CI) *P*-value**	**OR (95%CI) *P*-value**		**OR (95%CI) *P*-value**	**OR (95%CI) *P*-value**	

**Cotinine (Quartile)**			0.097			0.106			0.121
**Q1**	Reference	Reference		Reference	Reference		Reference	Reference	
**Q2**	3.510 (0.875, 14.088) 0.085	0.413 (0.142, 1.198) 0.112		**4.968 (1.149, 21.471) 0.038**	0.533 (0.175, 1.619) 0.274		4.193 (1.043, 16.853) 0.057	0.483 (0.141, 1.657) 0.261	
**Q3**	2.557 (0.478, 13.677) 0.279	0.668 (0.298, 1.497) 0.333		3.952 (0.737, 21.192) 0.117	0.903 (0.404, 2.018) 0.805		3.764 (0.678, 20.906) 0.145	0.834 (0.373, 1.867) 0.664	
**Q4**	1.459 (0.420, 5.064) 0.556	0.675 (0.333, 1.368) 0.282		3.118 (0.925, 10.515) 0.075	1.164 (0.622, 2.177) 0.638		2.763 (0.802, 9.521) 0.123	0.843 (0.402, 1.769) 0.657	
**Difficulty running errands alone**	**OR (95%CI) *P*-value**	**OR (95%CI) *P*-value**		**OR (95%CI) *P*-value**	**OR (95%CI) *P*-value**		**OR (95%CI) *P*-value**	**OR (95%CI) *P*-value**	
**Cotinine (Quartile)**			0.123			0.139			0.160
**Q1**	Reference	Reference		Reference	Reference		Reference	Reference	
**Q2**	2.620 (0.738, 9.302) 0.145	**0.516 (0.279, 0.954) 0.041**		3.375 (0.876, 13.008) 0.085	0.630 (0.331, 1.197) 0.167		2.725 (0.743, 9.998) 0.146	0.569 (0.289, 1.119) 0.118	
**Q3**	1.666 (0.265, 10.460) 0.589	1.168 (0.632, 2.157) 0.623		2.303 (0.352, 15.070) 0.390	1.516 (0.827, 2.779) 0.187		1.851 (0.260, 13.192) 0.546	1.387 (0.709, 2.714) 0.350	
**Q4**	3.083 (0.854, 11.135) 0.094	1.218 (0.735, 2.019) 0.449		**5.580 (1.514, 20.559) 0.014**	**1.913 (1.166, 3.140) 0.014**		3.706 (0.988, 13.900) 0.066	1.348 (0.745, 2.441) 0.336	
**Hearing difficulty**	**OR (95%CI) *P*-value**	**OR (95%CI) *P*-value**		**OR (95%CI) *P*-value**	**OR (95%CI) *P*-value**		**OR (95%CI) *P*-value**	**OR (95%CI) *P*-value**	
**Hydroxycotinine**	0.773 (0.406, 1.473) 0.438	0.450 (0.139, 1.457) 0.190	0.433	0.992 (0.603, 1.631) 0.974	0.597 (0.168, 2.119) 0.430	0.481	0.946 (0.561, 1.594) 0.835	0.563 (0.138, 2.298) 0.432	0.507
**Vision impairment**	**OR (95%CI) *P*-value**	**OR (95%CI) *P*-value**		**OR (95%CI) *P*-value**	**OR (95%CI) *P*-value**		**OR (95%CI) *P*-value**	**OR (95%CI) *P*-value**	
**Hydroxycotinine**	0.874 (0.473, 1.617) 0.671	0.937 (0.543, 1.617) 0.817	0.877	0.963 (0.533, 1.743) 0.902	1.085 (0.637, 1.849) 0.765	0.784	0.830 (0.409, 1.685) 0.611	0.783 (0.362, 1.692) 0.539	0.914
**Attention deficit**	**OR (95%CI) *P*-value**	**OR (95%CI) *P*-value**		**OR (95%CI) *P*-value**	**OR (95%CI) *P*-value**		**OR (95%CI) *P*-value**	**OR (95%CI) *P*-value**	

**hyperactivity disorder**									
**Hydroxycotinine**	0.846 (0.476, 1.506) 0.573	1.169 (0.796, 1.717) 0.429	0.332	0.879 (0.495, 1.563) 0.663	1.238 (0.849, 1.805) 0.274	0.302	0.735 (0.376, 1.435) 0.376	1.040 (0.659, 1.641) 0.866	0.408
**Walking difficulty**	**OR (95%CI) *P*-value**	**OR (95%CI) *P*-value**		**OR (95%CI) *P*-value**	**OR (95%CI) *P*-value**		**OR (95%CI) *P*-value**	**OR (95%CI) *P*-value**	
**Hydroxycotinine**	0.953 (0.605, 1.499) 0.835	1.487 (0.991, 2.230) 0.062	0.192	1.167 (0.768, 1.772) 0.473	**2.161 (1.457, 3.203) <0.001**	**0.048**	1.178 (0.775, 1.791) 0.450	**1.901 (1.115, 3.239) 0.027**	0.168
**Difficulty dressing or bathing independently**	**OR (95%CI) *P*-value**	**OR (95%CI) *P*-value**		**OR (95%CI) *P*-value**	**OR (95%CI) *P*-value**		**OR (95%CI) *P*-value**	**OR (95%CI) *P*-value**	
**Hydroxycotinine**	0.908 (0.478, 1.724) 0.769	0.930 (0.447, 1.936) 0.848	0.962	1.082 (0.560, 2.089) 0.816	1.207 (0.606, 2.407) 0.595	0.826	1.064 (0.499, 2.269) 0.874	1.062 (0.538, 2.095) 0.864	0.997
**Difficulty running errands alone**	**OR (95%CI) *P*-value**	**OR (95%CI) *P*-value**		**OR (95%CI) *P*-value**	**OR (95%CI) *P*-value**		**OR (95%CI) *P*-value**	**OR (95%CI) *P*-value**	
**Hydroxycotinine**	1.066 (0.605, 1.879) 0.826	1.352 (0.931, 1.964) 0.121	0.479	1.208 (0.652, 2.236) 0.551	**1.645 (1.138, 2.376) 0.011**	0.363	1.102 (0.518, 2.342) 0.803	1.315 (0.867, 1.996) 0.210	0.703
**Hearing difficulty**	**OR (95%CI) *P*-value**	**OR (95%CI) *P*-value**		**OR (95%CI) *P*-value**	**OR (95%CI) *P*-value**		**OR (95%CI) *P*-value**	**OR (95%CI) *P*-value**	
**Hydroxycotinine (Quartile)**			0.285			0.283			0.418
**Q1**	Reference	Reference		Reference	Reference		Reference	Reference	
**Q2**	2.025 (0.860, 4.768) 0.114	0.751 (0.349, 1.618) 0.470		1.900 (0.772, 4.676) 0.171	0.609 (0.276, 1.342) 0.226		1.828 (0.776, 4.306) 0.183	0.714 (0.309, 1.650) 0.440	
**Q3**	1.088 (0.449, 2.633) 0.853	0.571 (0.277, 1.174) 0.136		0.992 (0.393, 2.504) 0.987	0.551 (0.233, 1.302) 0.182		0.982 (0.411, 2.343) 0.967	0.519 (0.214, 1.262) 0.163	
**Q4**	0.990 (0.392, 2.499) 0.983	0.724 (0.338, 1.551) 0.412		1.624 (0.666, 3.956) 0.293	0.701 (0.304, 1.617) 0.410		1.632 (0.650, 4.096) 0.310	0.686 (0.289, 1.628) 0.403	

**Vision impairment**	**OR (95%CI) *P*-value**	**OR (95%CI) *P*-value**		**OR (95%CI) *P*-value**	**OR (95%CI) *P*-value**		**OR (95%CI) *P*-value**	**OR (95%CI) *P*-value**	
**Hydroxycotinine (Quartile)**			0.686			0.695			0.686
**Q1**	Reference	Reference		Reference	Reference		Reference	Reference	
**Q2**	0.896 (0.230, 3.488) 0.875	1.119 (0.596, 2.102) 0.728		0.824 (0.219, 3.100) 0.776	1.070 (0.568, 2.018) 0.834		0.834 (0.219, 3.182) 0.793	1.119 (0.589, 2.126) 0.735	
**Q3**	0.750 (0.208, 2.704) 0.663	1.652 (0.856, 3.188) 0.143		0.733 (0.196, 2.741) 0.647	1.748 (0.880, 3.471) 0.119		0.729 (0.220, 2.419) 0.612	1.616 (0.845, 3.088) 0.162	
**Q4**	0.784 (0.295, 2.085) 0.629	1.453 (0.675, 3.126) 0.346		0.927 (0.339, 2.531) 0.883	1.563 (0.705, 3.463) 0.278		0.672 (0.243, 1.858) 0.453	1.093 (0.460, 2.598) 0.842	
**Attention deficit hyperactivity disorder**	**OR (95%CI) *P*-value**	**OR (95%CI) *P*-value**		**OR (95%CI) *P*-value**	**OR (95%CI) *P*-value**		**OR (95%CI) *P*-value**	**OR (95%CI) *P*-value**	
**Hydroxycotinine (Quartile)**			0.152			0.178			0.067
**Q1**	Reference	Reference		Reference	Reference		Reference	Reference	
**Q2**	0.872 (0.356, 2.137) 0.766	1.571 (0.874, 2.824) 0.139		0.842 (0.345, 2.052) 0.707	1.551 (0.861, 2.791) 0.152		0.786 (0.323, 1.914) 0.602	1.562 (0.827, 2.953) 0.185	
**Q3**	1.493 (0.587, 3.800) 0.406	1.707 (0.879, 3.316) 0.123		1.488 (0.573, 3.867) 0.420	1.753 (0.915, 3.357) 0.099		1.318 (0.510, 3.405) 0.575	1.527 (0.784, 2.973) 0.228	
**Q4**	0.714 (0.324, 1.570) 0.407	**2.097 (1.154, 3.813) 0.020**		0.761 (0.340, 1.706) 0.512	**2.182 (1.218, 3.907) 0.012**		0.483 (0.209, 1.120) 0.105	1.779 (0.927, 3.417) 0.099	
**Walking difficulty**	**OR (95%CI) *P*-value**	**OR (95%CI) *P*-value**		**OR (95%CI) *P*-value**	**OR (95%CI) *P*-value**		**OR (95%CI) *P*-value**	**OR (95%CI) *P*-value**	
**Hydroxycotinine (Quartile)**			**0.017**			0.055			**0.033**
**Q1**	Reference	Reference		Reference	Reference		Reference	Reference	

**Q2**	**2.973 (1.192, 7.413) 0.025**	1.019 (0.593, 1.753) 0.945		**2.736 (1.056, 7.093) 0.045**	0.913 (0.536, 1.554) 0.739		2.677 (1.010, 7.099) 0.062	1.004 (0.613, 1.647) 0.986	
**Q3**	**4.321 (1.731, 10.786) 0.003**	1.111 (0.681, 1.813) 0.676		**4.531 (1.761, 11.655) 0.003**	1.213 (0.726, 2.025) 0.466		**4.814 (1.847, 12.546) 0.004**	1.113 (0.660, 1.877) 0.693	
**Q4**	2.275 (1.008, 5.137) 0.055	1.717 (0.973, 3.028) 0.070		**3.377 (1.451, 7.863) 0.008**	**2.084 (1.225, 3.547) 0.010**		**3.228 (1.364, 7.640) 0.015**	1.775 (0.991, 3.179) 0.068	
**Difficulty dressing or bathing independently**	**OR (95%CI) *P*-value**	**OR (95%CI) *P*-value**		**OR (95%CI) *P*-value**	**OR (95%CI) *P*-value**		**OR (95%CI) *P*-value**	**OR (95%CI) *P*-value**	
**Hydroxycotinine (Quartile)**			0.092			0.076			**0.037**
**Q1**	Reference	Reference		Reference	Reference		Reference	Reference	
**Q2**	0.362 (0.085, 1.545) 0.178	1.781 (0.832, 3.814) 0.145		0.316 (0.071, 1.396) 0.137	1.651 (0.725, 3.760) 0.240		0.275 (0.059, 1.278) 0.115	1.616 (0.616, 4.242) 0.341	
**Q3**	1.855 (0.460, 7.476) 0.390	0.989 (0.560, 1.745) 0.969		1.807 (0.414, 7.896) 0.436	1.043 (0.572, 1.900) 0.892		1.683 (0.374, 7.581) 0.505	0.883 (0.454, 1.716) 0.717	
**Q4**	0.780 (0.250, 2.434) 0.672	0.993 (0.529, 1.865) 0.982		1.050 (0.312, 3.529) 0.938	1.054 (0.542, 2.052) 0.877		0.891 (0.219, 3.627) 0.873	0.856 (0.393, 1.865) 0.699	
**Difficulty running errands alone**	**OR (95%CI) *P*-value**	**OR (95%CI) *P*-value**		**OR (95%CI) *P*-value**	**OR (95%CI) *P*-value**		**OR (95%CI) *P*-value**	**OR (95%CI) *P*-value**	
**Hydroxycotinine (Quartile)**			0.930			0.833			0.822
**Q1**	Reference	Reference		Reference	Reference		Reference	Reference	
**Q2**	1.376 (0.408, 4.640) 0.610	**1.946 (1.178, 3.214) 0.013**		1.249 (0.365, 4.278) 0.725	**1.855 (1.107, 3.106) 0.024**		1.124 (0.325, 3.889) 0.855	**1.885 (1.116, 3.183) 0.028**	
**Q3**	1.586 (0.326, 7.707) 0.571	**1.942 (1.084, 3.478) 0.032**		1.550 (0.297, 8.080) 0.606	**2.087 (1.164, 3.744) 0.018**		1.321 (0.245, 7.106) 0.749	1.753 (0.955, 3.216) 0.085	

**Q4**	2.364 (0.657, 8.500) 0.196	**2.384 (1.319, 4.309) 0.007**	3.000 (0.790, 11.399) 0.115	**2.637 (1.493, 4.658) 0.002**	2.082 (0.478, 9.073) 0.340	**2.072 (1.111, 3.862) 0.033**

Model 1: no covariates were adjusted.

Model 2: age was adjusted.

Model 3: age, body mass index, race/ethnicity, data release cycle, education level, marital status, income to poverty ratio, alcohol use, hypertension, diabetes, cancer, and cardiovascular diseases were adjusted.

Supplementary Table 4-2. Association between serum cotinine level, cotinine, hydroxycotinine and adverse health outcomes, stratified by age, Weighted.

	**Model 1**				**Model 2**				**Model 3**			
	**20-39 (n=929)**	**40-59 (n=624)**	**>=60 (n=617)**	***P*-interaction**	**20-39 (n=929)**	**40-59 (n=624)**	**>=60 (n=617)**	***P*-interaction**	**20-39 (n=929)**	**40-59 (n=624)**	**>=60 (n=617)**	***P*-interaction**
**Hearing difficulty**	**OR (95%CI) *P*-value**	**OR (95%CI) *P*-value**	**OR (95%CI) *P*-value**		**OR (95%CI) *P*-value**	**OR (95%CI) *P*-value**	**OR (95%CI) *P*-value**		**OR (95%CI) *P*-value**	**OR (95%CI) *P*-value**	**OR (95%CI) *P*-value**	
**Serum cotinine level**	0.807 (0.550, 1.184) 0.279	0.850 (0.604, 1.195) 0.355	1.051 (0.826, 1.336) 0.688	0.437	0.794 (0.541, 1.164) 0.244	0.825 (0.590, 1.154) 0.268	1.038 (0.829, 1.299) 0.749	0.378	0.813 (0.560, 1.181) 0.289	0.799 (0.537, 1.186) 0.277	1.048 (0.833, 1.318) 0.694	0.458
**Vision impairment**	**OR (95%CI) *P*-value**	**OR (95%CI) *P*-value**	**OR (95%CI) *P*-value**		**OR (95%CI) *P*-value**	**OR (95%CI) *P*-value**	**OR (95%CI) *P*-value**		**OR (95%CI) *P*-value**	**OR (95%CI) *P*-value**	**OR (95%CI) *P*-value**	
**Serum cotinine level**	1.022 (0.829, 1.260) 0.839	0.955 (0.748, 1.219) 0.714	1.044 (0.867, 1.257) 0.652	0.839	1.026 (0.834, 1.263) 0.808	0.963 (0.753, 1.232) 0.766	1.047 (0.868, 1.264) 0.634	0.855	0.996 (0.795, 1.249) 0.976	0.824 (0.613, 1.108) 0.213	0.929 (0.724, 1.192) 0.567	0.622
**Attention deficit hyperactivity disorder**	**OR (95%CI) *P*-value**	**OR (95%CI) *P*-value**	**OR (95%CI) *P*-value**		**OR (95%CI) *P*-value**	**OR (95%CI) *P*-value**	**OR (95%CI) *P*-value**		**OR (95%CI) *P*-value**	**OR (95%CI) *P*-value**	**OR (95%CI) *P*-value**	
**Serum cotinine level**	0.960 (0.829,	1.064 (0.875,	0.984 (0.806,	0.735	0.972 (0.838,	1.095 (0.899,	0.993 (0.813,	0.659	0.948 (0.819,	1.007 (0.795,	0.895 (0.723,	0.810

	1.113) 0.594	1.293) 0.540	1.201) 0.876		1.128) 0.713	1.334) 0.374	1.211) 0.943		1.098) 0.485	1.277) 0.953	1.108) 0.319	
**Walking difficulty**	**OR (95%CI) *P*-value**	**OR (95%CI) *P*-value**	**OR (95%CI) *P*-value**		**OR (95%CI) *P*-value**	**OR (95%CI) *P*-value**	**OR (95%CI) *P*-value**		**OR (95%CI) *P*-value**	**OR (95%CI) *P*-value**	**OR (95%CI) *P*-value**	
**Serum cotinine level**	1.025 (0.857, 1.226) 0.785	0.994 (0.818, 1.207) 0.948	**1.557 (1.194, 2.030) 0.002**	**0.014**	1.044 (0.870, 1.253) 0.649	1.033 (0.850, 1.255) 0.746	**1.594 (1.240, 2.049) <0.001**	**0.011**	1.038 (0.847, 1.273) 0.719	0.946 (0.710, 1.259) 0.706	**1.581 (1.235, 2.024) 0.002**	**0.016**
**Difficulty dressing or bathing independently**	**OR (95%CI) *P*-value**	**OR (95%CI) *P*-value**	**OR (95%CI) *P*-value**		**OR (95%CI) *P*-value**	**OR (95%CI) *P*-value**	**OR (95%CI) *P*-value**		**OR (95%CI) *P*-value**	**OR (95%CI) *P*-value**	**OR (95%CI) *P*-value**	
**Serum cotinine level**	1.053 (0.822, 1.349) 0.686	0.936 (0.737, 1.189) 0.591	1.148 (0.889, 1.483) 0.296	0.556	1.054 (0.820, 1.355) 0.683	0.938 (0.734, 1.198) 0.612	1.149 (0.890, 1.485) 0.294	0.565	1.056 (0.810, 1.376) 0.692	0.892 (0.670, 1.188) 0.443	1.098 (0.872, 1.383) 0.433	0.539
**Difficulty running errands alone**	**OR (95%CI) *P*-value**	**OR (95%CI) *P*-value**	**OR (95%CI) *P*-value**		**OR (95%CI) *P*-value**	**OR (95%CI) *P*-value**	**OR (95%CI) *P*-value**		**OR (95%CI) *P*-value**	**OR (95%CI) *P*-value**	**OR (95%CI) *P*-value**	
**Serum cotinine level**	1.010 (0.858, 1.188) 0.907	1.144 (0.965, 1.357) 0.130	1.140 (0.864, 1.505) 0.359	0.560	1.020 (0.859, 1.210) 0.824	1.171 (0.979, 1.399) 0.091	1.150 (0.875, 1.511) 0.322	0.496	0.992 (0.830, 1.187) 0.934	1.077 (0.885, 1.310) 0.466	1.060 (0.794, 1.414) 0.696	0.833
**Hearing difficulty**	**OR (95%CI) *P*-value**	**OR (95%CI) *P*-value**	**OR (95%CI) *P*-value**		**OR (95%CI) *P*-value**	**OR (95%CI) *P*-value**	**OR (95%CI) *P*-value**		**OR (95%CI) *P*-value**	**OR (95%CI) *P*-value**	**OR (95%CI) *P*-value**	
**Serum cotinine level (Quartile)**				0.206				0.190				0.368
**Q1**	Reference	Reference	Reference		Reference	Reference	Reference		Reference	Reference	Reference	

**Q2**	0.111 (0.009, 1.367) 0.095	**0.180 (0.058, 0.557) 0.005**	0.741 (0.320, 1.718) 0.490		0.113 (0.009, 1.401) 0.099	**0.165 (0.053, 0.520) 0.004**	0.752 (0.326, 1.735) 0.509		0.126 (0.010, 1.628) 0.132	**0.198 (0.062, 0.634) 0.015**	0.633 (0.256, 1.570) 0.339	
**Q3**	0.605 (0.047, 7.860) 0.703	0.553 (0.193, 1.587) 0.278	0.549 (0.246, 1.229) 0.154		0.611 (0.047, 7.999) 0.710	0.493 (0.163, 1.489) 0.219	0.568 (0.257, 1.256) 0.172		0.688 (0.051, 9.349) 0.782	0.627 (0.205, 1.918) 0.426	0.483 (0.209, 1.117) 0.108	
**Q4**	0.552 (0.086, 3.556) 0.536	0.675 (0.227, 2.009) 0.485	0.870 (0.375, 2.018) 0.747		0.520 (0.081, 3.359) 0.497	0.615 (0.203, 1.861) 0.396	0.908 (0.388, 2.127) 0.826		0.565 (0.080, 3.977) 0.575	0.660 (0.245, 1.780) 0.424	0.732 (0.283, 1.893) 0.530	
**Vision impairment**	**OR (95%CI) *P*-value**	**OR (95%CI) *P*-value**	**OR (95%CI) *P*-value**		**OR (95%CI) *P*-value**	**OR (95%CI) *P*-value**	**OR (95%CI) *P*-value**		**OR (95%CI) *P*-value**	**OR (95%CI) *P*-value**	**OR (95%CI) *P*-value**	
**Serum cotinine level (Quartile)**				0.968				0.971				0.936
**Q1**	Reference	Reference	Reference		Reference	Reference	Reference		Reference	Reference	Reference	
**Q2**	0.730 (0.108, 4.947) 0.749	1.229 (0.406, 3.727) 0.717	1.136 (0.374, 3.453) 0.823		0.727 (0.106, 4.966) 0.747	1.252 (0.407, 3.846) 0.698	1.132 (0.372, 3.439) 0.829		0.699 (0.104, 4.700) 0.718	1.295 (0.375, 4.469) 0.688	1.140 (0.352, 3.691) 0.830	
**Q3**	1.770 (0.430, 7.289) 0.435	1.282 (0.397, 4.139) 0.680	1.761 (0.560, 5.534) 0.340		1.765 (0.428, 7.275) 0.437	1.315 (0.393, 4.398) 0.660	1.745 (0.557, 5.467) 0.346		1.591 (0.402, 6.289) 0.518	1.134 (0.357, 3.606) 0.834	1.661 (0.469, 5.877) 0.443	
**Q4**	1.361 (0.344, 5.395) 0.663	1.121 (0.319, 3.932) 0.860	1.885 (0.711, 4.999) 0.211		1.380 (0.351, 5.428) 0.648	1.144 (0.324, 4.041) 0.836	1.867 (0.707, 4.929) 0.216		1.104 (0.284, 4.293) 0.888	0.717 (0.197, 2.611) 0.621	1.100 (0.317, 3.819) 0.882	

**Attention deficit hyperactivity disorder**	**OR (95%CI) *P*-value**	**OR (95%CI) *P*-value**	**OR (95%CI) *P*-value**		**OR (95%CI) *P*-value**	**OR (95%CI) *P*-value**	**OR (95%CI) *P*-value**		**OR (95%CI) *P*-value**	**OR (95%CI) *P*-value**	**OR (95%CI) *P*-value**	
**Serum cotinine level (Quartile)**				0.456				0.516				0.333
**Q1**	Reference	Reference	Reference		Reference	Reference	Reference		Reference	Reference	Reference	
**Q2**	0.712 (0.300, 1.688) 0.446	0.950 (0.429, 2.104) 0.901	1.196 (0.494, 2.896) 0.694		0.703 (0.291, 1.698) 0.439	1.002 (0.448, 2.242) 0.996	1.182 (0.486, 2.874) 0.715		0.695 (0.295, 1.637) 0.417	1.059 (0.472, 2.378) 0.890	1.105 (0.474, 2.572) 0.820	
**Q3**	1.144 (0.439, 2.976) 0.785	1.542 (0.736, 3.231) 0.259	1.895 (0.782, 4.589) 0.166		1.135 (0.435, 2.964) 0.798	1.665 (0.786, 3.529) 0.192	1.849 (0.754, 4.536) 0.189		1.045 (0.424, 2.579) 0.925	1.590 (0.699, 3.617) 0.286	1.722 (0.725, 4.093) 0.236	
**Q4**	0.875 (0.443, 1.727) 0.703	1.785 (0.679, 4.690) 0.248	**3.028 (1.283, 7.145) 0.016**		0.911 (0.461, 1.802) 0.791	1.901 (0.727, 4.970) 0.199	**2.953 (1.256, 6.942) 0.018**		0.712 (0.387, 1.312) 0.292	1.558 (0.536, 4.532) 0.428	2.339 (1.046, 5.227) 0.055	
**Walking difficulty**	**OR (95%CI) *P*-value**	**OR (95%CI) *P*-value**	**OR (95%CI) *P*-value**		**OR (95%CI) *P*-value**	**OR (95%CI) *P*-value**	**OR (95%CI) *P*-value**		**OR (95%CI) *P*-value**	**OR (95%CI) *P*-value**	**OR (95%CI) *P*-value**	
**Serum cotinine level (Quartile)**				**0.012**				**0.012**				**0.008**
**Q1**	Reference	Reference	Reference		Reference	Reference	Reference		Reference	Reference	Reference	
**Q2**	2.625 (0.642, 10.730) 0.188	0.532 (0.172, 1.641) 0.280	1.359 (0.589, 3.133) 0.477		2.591 (0.628, 10.690) 0.197	0.570 (0.186, 1.748) 0.332	1.340 (0.573, 3.135) 0.505		2.746 (0.668, 11.285) 0.180	0.699 (0.240, 2.039) 0.522	1.430 (0.650, 3.145) 0.387	
**Q3**	1.428 (0.336,	2.477 (0.953,	1.022 (0.593,		1.413 (0.328,	**2.771 (1.067,**	0.985 (0.563,		1.339 (0.313,	2.865 (1.036,	0.879 (0.511,	

	6.073) 0.633	6.441) 0.071	1.763) 0.937		6.091) 0.646	**7.196) 0.044**	1.723) 0.957		5.726) 0.699	7.921) 0.059	1.513) 0.648	
**Q4**	1.493 (0.514, 4.332) 0.466	2.022 (0.753, 5.429) 0.171	**3.361 (1.720, 6.569) 0.001**		1.579 (0.540, 4.623) 0.410	2.211 (0.845, 5.786) 0.115	**3.283 (1.702, 6.334) 0.001**		1.394 (0.472, 4.115) 0.556	2.033 (0.765, 5.403) 0.174	**2.720 (1.453, 5.093) 0.006**	
**Difficulty dressing or bathing independently**	**OR (95%CI) *P*-value**	**OR (95%CI) *P*-value**	**OR (95%CI) *P*-value**		**OR (95%CI) *P*-value**	**OR (95%CI) *P*-value**	**OR (95%CI) *P*-value**		**OR (95%CI) *P*-value**	**OR (95%CI) *P*-value**	**OR (95%CI) *P*-value**	
**Serum cotinine level (Quartile)**				0.120				0.091				0.074
**Q1**	Reference	Reference	Reference		Reference	Reference	Reference		Reference	Reference	Reference	
**Q2**	1.060 (0.131, 8.564) 0.957	1.104 (0.314, 3.883) 0.879	0.817 (0.279, 2.394) 0.715		1.057 (0.131, 8.539) 0.959	1.117 (0.314, 3.975) 0.866	0.815 (0.274, 2.421) 0.714		1.072 (0.126, 9.139) 0.950	1.036 (0.300, 3.576) 0.956	0.740 (0.261, 2.099) 0.579	
**Q3**	0.496 (0.056, 4.383) 0.533	4.402 (1.223, 15.843) 0.030	0.575 (0.227, 1.456) 0.251		0.495 (0.056, 4.370) 0.532	**4.474 (1.286, 15.561) 0.025**	0.572 (0.225, 1.456) 0.249		0.425 (0.048, 3.786) 0.454	**4.267 (1.123, 16.219) 0.049**	0.483 (0.182, 1.281) 0.163	
**Q4**	0.795 (0.111, 5.707) 0.821	2.249 (0.660, 7.659) 0.204	1.018 (0.455, 2.280) 0.965		0.802 (0.110, 5.826) 0.829	2.279 (0.662, 7.844) 0.200	1.012 (0.445, 2.301) 0.978		0.698 (0.083, 5.876) 0.745	1.771 (0.522, 6.007) 0.373	0.823 (0.360, 1.884) 0.651	
**Difficulty running errands alone**	**OR (95%CI) *P*-value**	**OR (95%CI) *P*-value**	**OR (95%CI) *P*-value**		**OR (95%CI) *P*-value**	**OR (95%CI) *P*-value**	**OR (95%CI) *P*-value**		**OR (95%CI) *P*-value**	**OR (95%CI) *P*-value**	**OR (95%CI) *P*-value**	

**Serum cotinine level (Quartile)**				0.504				0.416				0.257
**Q1**	Reference	Reference	Reference		Reference	Reference	Reference		Reference	Reference	Reference	
**Q2**	0.566 (0.161, 1.999) 0.383	1.164 (0.309, 4.389) 0.824	0.760 (0.339, 1.703) 0.510		0.561 (0.157, 1.999) 0.379	1.217 (0.315, 4.702) 0.777	0.751 (0.336, 1.680) 0.491		0.579 (0.165, 2.030) 0.406	1.219 (0.360, 4.131) 0.755	0.630 (0.262, 1.514) 0.317	
**Q3**	1.415 (0.312, 6.419) 0.655	**3.786 (1.504, 9.531) 0.008**	1.075 (0.469, 2.467) 0.865		1.406 (0.310, 6.385) 0.662	**4.041 (1.610, 10.143) 0.005**	1.051 (0.456, 2.420) 0.908		1.261 (0.277, 5.743) 0.768	**3.796 (1.308, 11.017) 0.026**	0.749 (0.312, 1.803) 0.529	
**Q4**	2.161 (0.575, 8.124) 0.262	**3.835 (1.353, 10.872) 0.016**	1.682 (0.760, 3.723) 0.208		2.240 (0.588, 8.538) 0.246	**4.045 (1.405, 11.641) 0.014**	1.643 (0.740, 3.647) 0.231		1.765 (0.426, 7.316) 0.445	**2.966 (1.198, 7.345) 0.032**	1.177 (0.506, 2.738) 0.710	
**Hearing difficulty**	**OR (95%CI) *P*-value**	**OR (95%CI) *P*-value**	**OR (95%CI) *P*-value**		**OR (95%CI) *P*-value**	**OR (95%CI) *P*-value**	**OR (95%CI) *P*-value**		**OR (95%CI) *P*-value**	**OR (95%CI) *P*-value**	**OR (95%CI) *P*-value**	
**Cotinine**	0.770 (0.461, 1.288) 0.326	0.820 (0.502, 1.339) 0.432	0.968 (0.678, 1.381) 0.857	0.740	0.755 (0.451, 1.263) 0.290	0.782 (0.478, 1.278) 0.332	0.953 (0.676, 1.342) 0.783	0.698	0.782 (0.471, 1.296) 0.350	0.761 (0.416, 1.395) 0.387	0.984 (0.704, 1.375) 0.927	0.723
**Vision impairment**	**OR (95%CI) *P*-value**	**OR (95%CI) *P*-value**	**OR (95%CI) *P*-value**		**OR (95%CI) *P*-value**	**OR (95%CI) *P*-value**	**OR (95%CI) *P*-value**		**OR (95%CI) *P*-value**	**OR (95%CI) *P*-value**	**OR (95%CI) *P*-value**	
**Cotinine**	1.032 (0.780, 1.365) 0.826	0.974 (0.704, 1.347) 0.873	1.005 (0.744, 1.356) 0.976	0.970	1.037 (0.786, 1.368) 0.799	0.986 (0.709, 1.371) 0.933	1.008 (0.745, 1.364) 0.959	0.978	0.992 (0.733, 1.341) 0.957	0.799 (0.533, 1.199) 0.290	0.835 (0.519, 1.343) 0.465	0.710
**Attention deficit**	**OR (95%CI) *P*-value**	**OR (95%CI) *P*-value**	**OR (95%CI) *P*-value**		**OR (95%CI) *P*-value**	**OR (95%CI) *P*-value**	**OR (95%CI) *P*-value**		**OR (95%CI) *P*-value**	**OR (95%CI) *P*-value**	**OR (95%CI) *P*-value**	

**hyperactivity disorder**												
**Cotinine**	0.940 (0.775, 1.140) 0.535	1.117 (0.853, 1.462) 0.426	0.927 (0.680, 1.264) 0.635	0.614	0.954 (0.787, 1.157) 0.633	1.165 (0.891, 1.523) 0.272	0.937 (0.690, 1.272) 0.677	0.512	0.922 (0.762, 1.115) 0.411	1.051 (0.762, 1.451) 0.764	0.817 (0.573, 1.165) 0.277	0.673
**Walking difficulty**	**OR (95%CI) *P*-value**	**OR (95%CI) *P*-value**	**OR (95%CI) *P*-value**		**OR (95%CI) *P*-value**	**OR (95%CI) *P*-value**	**OR (95%CI) *P*-value**		**OR (95%CI) *P*-value**	**OR (95%CI) *P*-value**	**OR (95%CI) *P*-value**	
**Cotinine**	1.026 (0.808, 1.302) 0.836	0.983 (0.765, 1.263) 0.894	**1.727 (1.118, 2.668) 0.018**	0.076	1.046 (0.822, 1.332) 0.717	1.039 (0.812, 1.330) 0.762	**1.777 (1.166, 2.706) 0.011**	0.072	1.036 (0.790, 1.359) 0.799	0.919 (0.626, 1.347) 0.668	**1.796 (1.207, 2.670) 0.008**	**0.046**
**Difficulty dressing or bathing independently**	**OR (95%CI) *P*-value**	**OR (95%CI) *P*-value**	**OR (95%CI) *P*-value**		**OR (95%CI) *P*-value**	**OR (95%CI) *P*-value**	**OR (95%CI) *P*-value**		**OR (95%CI) *P*-value**	**OR (95%CI) *P*-value**	**OR (95%CI) *P*-value**	
**Cotinine**	1.046 (0.754, 1.450) 0.791	0.960 (0.707, 1.303) 0.796	1.193 (0.844, 1.687) 0.324	0.685	1.047 (0.753, 1.456) 0.787	0.964 (0.708, 1.312) 0.815	1.194 (0.844, 1.690) 0.322	0.692	1.042 (0.738, 1.471) 0.817	0.901 (0.629, 1.292) 0.578	1.147 (0.830, 1.584) 0.416	0.638
**Difficulty running errands alone**	**OR (95%CI) *P*-value**	**OR (95%CI) *P*-value**	**OR (95%CI) *P*-value**		**OR (95%CI) *P*-value**	**OR (95%CI) *P*-value**	**OR (95%CI) *P*-value**		**OR (95%CI) *P*-value**	**OR (95%CI) *P*-value**	**OR (95%CI) *P*-value**	
**Cotinine**	0.960 (0.793, 1.162) 0.679	1.246 (0.999, 1.554) 0.058	1.178 (0.789, 1.758) 0.428	0.241	0.971 (0.796, 1.185) 0.772	**1.287 (1.024, 1.617) 0.036**	1.188 (0.800, 1.762) 0.398	0.178	0.928 (0.752, 1.144) 0.491	1.152 (0.890, 1.492) 0.294	1.083 (0.702, 1.673) 0.721	0.466
**Hearing difficulty**	**OR (95%CI) *P*-value**	**OR (95%CI) *P*-value**	**OR (95%CI) *P*-value**		**OR (95%CI) *P*-value**	**OR (95%CI) *P*-value**	**OR (95%CI) *P*-value**		**OR (95%CI) *P*-value**	**OR (95%CI) *P*-value**	**OR (95%CI) *P*-value**	

**Cotinine (Quartile)**					0.936				0.946				0.964
	**Q1**	Reference	Reference	Reference		Reference	Reference	Reference		Reference	Reference	Reference	
	**Q2**	0.106 (0.008, 1.344) 0.092	0.380 (0.111, 1.305) 0.133	**0.397 (0.175, 0.900) 0.034**		0.102 (0.008, 1.308) 0.089	0.354 (0.102, 1.231) 0.112	**0.387 (0.166, 0.904) 0.035**		0.108 (0.008, 1.385) 0.107	0.368 (0.108, 1.257) 0.130	**0.301 (0.119, 0.756) 0.021**	
	**Q3**	0.550 (0.042, 7.172) 0.651	0.607 (0.191, 1.928) 0.403	0.427 (0.187, 0.975) 0.051		0.531 (0.040, 7.008) 0.634	0.534 (0.158, 1.806) 0.320	0.440 (0.193, 1.001) 0.059		0.598 (0.043, 8.265) 0.706	0.665 (0.194, 2.279) 0.526	0.416 (0.161, 1.077) 0.090	
	**Q4**	0.510 (0.077, 3.389) 0.490	0.728 (0.225, 2.359) 0.600	0.738 (0.377, 1.445) 0.382		0.462 (0.069, 3.100) 0.432	0.663 (0.199, 2.203) 0.507	0.746 (0.374, 1.485) 0.410		0.491 (0.065, 3.680) 0.499	0.677 (0.232, 1.975) 0.486	0.574 (0.275, 1.197) 0.158	
**Vision impairment**		**OR (95%CI) *P*-value**	**OR (95%CI) *P*-value**	**OR (95%CI) *P*-value**		**OR (95%CI) *P*-value**	**OR (95%CI) *P*-value**	**OR (95%CI) *P*-value**		**OR (95%CI) *P*-value**	**OR (95%CI) *P*-value**	**OR (95%CI) *P*-value**	
**Cotinine (Quartile)**					0.553				0.582				0.758
	**Q1**	Reference	Reference	Reference		Reference	Reference	Reference		Reference	Reference	Reference	
	**Q2**	0.682 (0.141, 3.308) 0.638	1.257 (0.389, 4.066) 0.705	0.716 (0.266, 1.924) 0.512		0.688 (0.141, 3.347) 0.646	1.276 (0.391, 4.165) 0.689	0.718 (0.266, 1.936) 0.517		0.643 (0.139, 2.973) 0.580	1.254 (0.350, 4.498) 0.733	0.614 (0.228, 1.652) 0.348	
	**Q3**	2.365 (0.478, 11.695) 0.299	1.434 (0.419, 4.902) 0.569	1.349 (0.481, 3.783) 0.573		2.383 (0.481, 11.797) 0.295	1.476 (0.414, 5.255) 0.552	1.337 (0.476, 3.756) 0.585		2.113 (0.429, 10.416) 0.372	1.205 (0.346, 4.201) 0.773	1.396 (0.413, 4.726) 0.599	
	**Q4**	1.207 (0.266, 5.475) 0.809	1.119 (0.309, 4.052) 0.865	1.855 (0.716, 4.808) 0.212		1.234 (0.274, 5.558) 0.786	1.142 (0.313, 4.174) 0.842	1.850 (0.712, 4.803) 0.215		0.952 (0.213, 4.250) 0.950	0.694 (0.181, 2.657) 0.601	0.992 (0.288, 3.415) 0.990	

**Attention deficit hyperactivity disorder**	**OR (95%CI) *P*-value**	**OR (95%CI) *P*-value**	**OR (95%CI) *P*-value**		**OR (95%CI) *P*-value**	**OR (95%CI) *P*-value**	**OR (95%CI) *P*-value**		**OR (95%CI) *P*-value**	**OR (95%CI) *P*-value**	**OR (95%CI) *P*-value**	
**Cotinine (Quartile)**				0.633				0.662				0.686
**Q1**	Reference	Reference	Reference		Reference	Reference	Reference		Reference	Reference	Reference	
**Q2**	0.961 (0.378, 2.446) 0.934	0.900 (0.339, 2.390) 0.834	1.301 (0.604, 2.799) 0.506		0.986 (0.385, 2.527) 0.977	0.941 (0.347, 2.557) 0.906	1.313 (0.610, 2.827) 0.491		0.910 (0.326, 2.544) 0.860	0.914 (0.338, 2.472) 0.861	1.372 (0.645, 2.919) 0.423	
**Q3**	1.084 (0.388, 3.025) 0.878	1.606 (0.684, 3.767) 0.284	1.728 (0.715, 4.178) 0.233		1.110 (0.398, 3.094) 0.844	1.757 (0.742, 4.162) 0.209	1.686 (0.689, 4.122) 0.260		1.040 (0.381, 2.839) 0.941	1.583 (0.578, 4.338) 0.385	1.783 (0.689, 4.612) 0.251	
**Q4**	1.057 (0.511, 2.190) 0.881	1.688 (0.616, 4.627) 0.316	**2.932 (1.274, 6.744) 0.016**		1.134 (0.547, 2.350) 0.738	1.802 (0.659, 4.926) 0.259	**2.919 (1.288, 6.616) 0.015**		0.863 (0.407, 1.830) 0.705	1.442 (0.471, 4.422) 0.531	2.206 (0.973, 5.004) 0.076	
**Walking difficulty**	**OR (95%CI) *P*-value**	**OR (95%CI) *P*-value**	**OR (95%CI) *P*-value**		**OR (95%CI) *P*-value**	**OR (95%CI) *P*-value**	**OR (95%CI) *P*-value**		**OR (95%CI) *P*-value**	**OR (95%CI) *P*-value**	**OR (95%CI) *P*-value**	
**Cotinine (Quartile)**				**0.043**				**0.03**				**0.045**
**Q1**	Reference	Reference	Reference		Reference	Reference	Reference		Reference	Reference	Reference	
**Q2**	3.143 (0.704, 14.020) 0.143	0.643 (0.232, 1.777) 0.400	0.900 (0.353, 2.296) 0.826		3.262 (0.733, 14.515) 0.130	0.681 (0.245, 1.895) 0.467	0.909 (0.356, 2.319) 0.843		3.817 (0.845, 17.237) 0.101	0.690 (0.263, 1.809) 0.461	0.839 (0.316, 2.225) 0.729	
**Q3**	1.535 (0.330, 7.152) 0.589	2.408 (0.925, 6.270) 0.081	1.166 (0.638, 2.131) 0.621		1.584 (0.338, 7.420) 0.563	**2.742 (1.065, 7.055) 0.044**	1.125 (0.609, 2.078) 0.710		1.590 (0.339, 7.461) 0.565	2.766 (0.993, 7.701) 0.069	1.165 (0.605, 2.243) 0.654	

**Q4**	1.628 (0.509, 5.213) 0.417	1.941 (0.760, 4.962) 0.175	**2.687 (1.471, 4.910) 0.003**		1.789 (0.560, 5.717) 0.334	2.128 (0.864, 5.241) 0.110	**2.697 (1.518, 4.794) 0.002**		1.642 (0.505, 5.344) 0.422	1.786 (0.705, 4.528) 0.239	2.006 (0.995, 4.044) 0.070	
**Difficulty dressing or bathing independently**	**OR (95%CI) *P*-value**	**OR (95%CI) *P*-value**	**OR (95%CI) *P*-value**		**OR (95%CI) *P*-value**	**OR (95%CI) *P*-value**	**OR (95%CI) *P*-value**		**OR (95%CI) *P*-value**	**OR (95%CI) *P*-value**	**OR (95%CI) *P*-value**	
**Cotinine (Quartile)**				**0.018**				**0.012**				**0.007**
**Q1**	Reference	Reference	Reference		Reference	Reference	Reference		Reference	Reference	Reference	
**Q2**	**16.995 (1.711, 168.802) 0.021**	0.613 (0.186, 2.024) 0.428	0.891 (0.263, 3.012) 0.853		**17.110 (1.734, 168.819) 0.021**	0.620 (0.187, 2.053) 0.440	0.893 (0.265, 3.007) 0.856		**18.521 (1.931, 177.606) 0.022**	0.518 (0.157, 1.710) 0.297	0.720 (0.220, 2.351) 0.593	
**Q3**	4.156 (0.384, 44.935) 0.249	**3.754 (1.168, 12.070) 0.033**	0.521 (0.194, 1.403) 0.206		4.181 (0.387, 45.160) 0.247	**3.839 (1.259, 11.710) 0.024**	0.518 (0.192, 1.397) 0.202		3.776 (0.345, 41.345) 0.293	3.425 (0.978, 11.998) 0.072	0.476 (0.178, 1.269) 0.157	
**Q4**	6.753 (0.762, 59.880) 0.095	1.761 (0.573, 5.412) 0.330	1.308 (0.598, 2.858) 0.506		6.873 (0.783, 60.342) 0.091	1.789 (0.580, 5.521) 0.319	1.305 (0.595, 2.860) 0.511		6.006 (0.662, 54.494) 0.131	1.341 (0.452, 3.976) 0.604	0.985 (0.420, 2.307) 0.972	
**Difficulty running errands alone**	**OR (95%CI) *P*-value**	**OR (95%CI) *P*-value**	**OR (95%CI) *P*-value**		**OR (95%CI) *P*-value**	**OR (95%CI) *P*-value**	**OR (95%CI) *P*-value**		**OR (95%CI) *P*-value**	**OR (95%CI) *P*-value**	**OR (95%CI) *P*-value**	
**Cotinine (Quartile)**				0.360				0.241				0.297
**Q1**	Reference	Reference	Reference		Reference	Reference	Reference		Reference	Reference	Reference	
**Q2**	1.805 (0.471,	1.015 (0.307,	0.980 (0.452,		1.846 (0.479,	1.055 (0.314,	0.987 (0.455,		1.786 (0.475,	0.907 (0.315,	0.840 (0.367,	

	6.912) 0.395	3.354) 0.981	2.125) 0.959		7.113) 0.379	3.539) 0.932	2.140) 0.974		6.721) 0.404	2.610) 0.858	1.923) 0.686	
**Q3**	2.228 (0.511, 9.714) 0.294	**3.622 (1.608, 8.156) 0.004**	1.055 (0.504, 2.209) 0.888		2.274 (0.523, 9.889) 0.281	**3.912 (1.766, 8.665) 0.002**	1.031 (0.494, 2.151) 0.936		2.051 (0.471, 8.928) 0.353	**3.392 (1.176, 9.784) 0.038**	0.895 (0.404, 1.984) 0.788	
**Q4**	**3.874 (1.150, 13.049) 0.036**	**3.457 (1.363, 8.772) 0.013**	1.675 (0.750, 3.739) 0.216		**4.117 (1.227, 13.816) 0.028**	**3.655 (1.426, 9.368) 0.011**	1.664 (0.744, 3.722) 0.224		3.161 (0.915, 10.917) 0.088	**2.512 (1.170, 5.392) 0.031**	1.113 (0.458, 2.705) 0.815	
**Hearing difficulty**	**OR (95%CI) *P*-value**	**OR (95%CI) *P*-value**	**OR (95%CI) *P*-value**		**OR (95%CI) *P*-value**	**OR (95%CI) *P*-value**	**OR (95%CI) *P*-value**		**OR (95%CI) *P*-value**	**OR (95%CI) *P*-value**	**OR (95%CI) *P*-value**	
**Hydroxycotinine**	0.387 (0.080, 1.872) 0.245	0.553 (0.206, 1.487) 0.248	1.510 (0.827, 2.756) 0.187	0.129	0.364 (0.075, 1.760) 0.216	0.517 (0.195, 1.368) 0.192	1.446 (0.833, 2.511) 0.198	0.098	0.389 (0.082, 1.836) 0.246	0.443 (0.163, 1.206) 0.125	1.403 (0.760, 2.591) 0.291	0.152
**Vision impairment**	**OR (95%CI) *P*-value**	**OR (95%CI) *P*-value**	**OR (95%CI) *P*-value**		**OR (95%CI) *P*-value**	**OR (95%CI) *P*-value**	**OR (95%CI) *P*-value**		**OR (95%CI) *P*-value**	**OR (95%CI) *P*-value**	**OR (95%CI) *P*-value**	
**Hydroxycotinine**	1.050 (0.572, 1.928) 0.877	0.742 (0.286, 1.929) 0.544	1.306 (0.800, 2.133) 0.292	0.545	1.066 (0.580, 1.957) 0.838	0.756 (0.291, 1.967) 0.570	1.322 (0.805, 2.171) 0.276	0.550	1.014 (0.504, 2.039) 0.970	0.467 (0.137, 1.599) 0.238	1.002 (0.579, 1.734) 0.994	0.499
**Attention deficit hyperactivity disorder**	**OR (95%CI) *P*-value**	**OR (95%CI) *P*-value**	**OR (95%CI) *P*-value**		**OR (95%CI) *P*-value**	**OR (95%CI) *P*-value**	**OR (95%CI) *P*-value**		**OR (95%CI) *P*-value**	**OR (95%CI) *P*-value**	**OR (95%CI) *P*-value**	
**Hydroxycotinine**	0.927 (0.549, 1.565) 0.778	1.066 (0.574, 1.978) 0.841	1.115 (0.656, 1.898) 0.689	0.876	0.972 (0.570, 1.657) 0.918	1.142 (0.610, 2.139) 0.680	1.154 (0.674, 1.975) 0.604	0.884	0.910 (0.522, 1.587) 0.744	0.862 (0.401, 1.850) 0.706	0.824 (0.471, 1.441) 0.504	0.968

**Walking difficulty**	**OR (95%CI) *P*-value**	**OR (95%CI) *P*-value**	**OR (95%CI) *P*-value**		**OR (95%CI) *P*-value**	**OR (95%CI) *P*-value**	**OR (95%CI) *P*-value**		**OR (95%CI) *P*-value**	**OR (95%CI) *P*-value**	**OR (95%CI) *P*-value**	
**Hydroxycotinine**	1.116 (0.641, 1.943) 0.700	1.012 (0.529, 1.939) 0.971	**4.660 (1.927, 11.268) 0.002**	**0.009**	1.201 (0.679, 2.121) 0.533	1.118 (0.582, 2.146) 0.739	**4.898 (2.258, 10.627) <0.001**	**0.005**	1.205 (0.645, 2.251) 0.565	0.882 (0.341, 2.285) 0.799	**4.568 (1.916, 10.891) 0.003**	**0.025**
**Difficulty dressing or bathing independently**	**OR (95%CI) *P*-value**	**OR (95%CI) *P*-value**	**OR (95%CI) *P*-value**		**OR (95%CI) *P*-value**	**OR (95%CI) *P*-value**	**OR (95%CI) *P*-value**		**OR (95%CI) *P*-value**	**OR (95%CI) *P*-value**	**OR (95%CI) *P*-value**	
**Hydroxycotinine**	1.279 (0.653, 2.504) 0.477	0.641 (0.243, 1.686) 0.373	1.482 (0.733, 2.999) 0.280	0.396	1.285 (0.645, 2.561) 0.480	0.644 (0.239, 1.738) 0.390	1.488 (0.736, 3.011) 0.275	0.400	1.351 (0.628, 2.904) 0.450	0.551 (0.167, 1.821) 0.339	1.237 (0.657, 2.332) 0.517	0.39
**Difficulty running errands alone**	**OR (95%CI) *P*-value**	**OR (95%CI) *P*-value**	**OR (95%CI) *P*-value**		**OR (95%CI) *P*-value**	**OR (95%CI) *P*-value**	**OR (95%CI) *P*-value**		**OR (95%CI) *P*-value**	**OR (95%CI) *P*-value**	**OR (95%CI) *P*-value**	
**Hydroxycotinine**	1.266 (0.742, 2.160) 0.391	1.226 (0.686, 2.191) 0.495	1.481 (0.729, 3.008) 0.284	0.925	1.320 (0.741, 2.352) 0.352	1.299 (0.706, 2.391) 0.406	1.531 (0.761, 3.080) 0.240	0.939	1.286 (0.703, 2.354) 0.423	1.000 (0.518, 1.933) 0.999	1.172 (0.579, 2.375) 0.664	0.846
**Hearing difficulty**	**OR (95%CI) *P*-value**	**OR (95%CI) *P*-value**	**OR (95%CI) *P*-value**		**OR (95%CI) *P*-value**	**OR (95%CI) *P*-value**	**OR (95%CI) *P*-value**		**OR (95%CI) *P*-value**	**OR (95%CI) *P*-value**	**OR (95%CI) *P*-value**	
**Hydroxycotinine (Quartile)**				0.398				0.364				0.497
**Q1**	Reference	Reference	Reference		Reference	Reference	Reference		Reference	Reference	Reference	
**Q2**	2.449 (0.209,	0.341 (0.088,	1.152 (0.518,		2.609 (0.228,	0.321 (0.080,	1.164 (0.530,		2.863 (0.252,	0.358 (0.089,	1.327 (0.601,	

		28.634) 0.480	1.317) 0.128	2.565) 0.731		29.877) 0.446	1.298) 0.120	2.558) 0.707		32.566) 0.409	1.444) 0.168	2.934) 0.494	
	**Q3**	2.175 (0.219, 21.570) 0.511	**0.332 (0.136, 0.808) 0.020**	0.821 (0.449, 1.501) 0.526		2.259 (0.230, 22.240) 0.490	**0.314 (0.129, 0.767) 0.016**	0.803 (0.434, 1.485) 0.489		2.330 (0.219, 24.832) 0.494	**0.373 (0.156, 0.891) 0.041**	0.739 (0.384, 1.422) 0.378	
	**Q4**	1.538 (0.228, 10.378) 0.661	0.740 (0.271, 2.019) 0.560	1.165 (0.539, 2.520) 0.700		1.502 (0.223, 10.113) 0.679	0.695 (0.248, 1.949) 0.494	1.247 (0.560, 2.776) 0.593		1.643 (0.225, 12.012) 0.631	0.700 (0.274, 1.793) 0.468	1.157 (0.451, 2.971) 0.765	
**Vision impairment**		**OR (95%CI) *P*-value**	**OR (95%CI) *P*-value**	**OR (95%CI) *P*-value**		**OR (95%CI) *P*-value**	**OR (95%CI) *P*-value**	**OR (95%CI) *P*-value**		**OR (95%CI) *P*-value**	**OR (95%CI) *P*-value**	**OR (95%CI) *P*-value**	
**Hydroxycotinine (Quartile)**					0.685				0.691				0.805
	**Q1**	Reference	Reference	Reference		Reference	Reference	Reference		Reference	Reference	Reference	
	**Q2**	0.925 (0.336, 2.547) 0.881	1.085 (0.311, 3.783) 0.899	0.935 (0.262, 3.331) 0.918		0.913 (0.331, 2.515) 0.861	1.097 (0.308, 3.910) 0.887	0.933 (0.260, 3.352) 0.916		0.959 (0.345, 2.669) 0.937	1.032 (0.287, 3.717) 0.962	1.065 (0.282, 4.029) 0.927	
	**Q3**	0.876 (0.383, 2.004) 0.756	1.654 (0.522, 5.244) 0.398	1.380 (0.537, 3.547) 0.508		0.870 (0.383, 1.976) 0.741	1.672 (0.525, 5.325) 0.391	1.385 (0.540, 3.552) 0.502		0.853 (0.375, 1.942) 0.710	1.490 (0.468, 4.745) 0.509	1.429 (0.574, 3.559) 0.454	
	**Q4**	1.067 (0.389, 2.930) 0.900	0.953 (0.292, 3.108) 0.937	2.000 (0.758, 5.275) 0.170		1.073 (0.392, 2.937) 0.892	0.965 (0.295, 3.159) 0.954	1.975 (0.738, 5.282) 0.184		0.939 (0.331, 2.661) 0.907	0.588 (0.173, 1.996) 0.407	1.302 (0.376, 4.510) 0.682	
**Attention deficit hyperactivity disorder**		**OR (95%CI) *P*-value**	**OR (95%CI) *P*-value**	**OR (95%CI) *P*-value**		**OR (95%CI) *P*-value**	**OR (95%CI) *P*-value**	**OR (95%CI) *P*-value**		**OR (95%CI) *P*-value**	**OR (95%CI) *P*-value**	**OR (95%CI) *P*-value**	

**Hydroxycotinine (Quartile)**				**0.004**				**0.009**				**0.004**
**Q1**	Reference	Reference	Reference		Reference	Reference	Reference		Reference	Reference	Reference	
**Q2**	1.881 (0.823, 4.300) 0.143	1.106 (0.457, 2.676) 0.825	0.884 (0.405, 1.930) 0.759		1.814 (0.790, 4.166) 0.170	1.143 (0.467, 2.798) 0.772	0.879 (0.413, 1.872) 0.741		1.864 (0.796, 4.363) 0.171	0.943 (0.331, 2.688) 0.913	0.989 (0.477, 2.051) 0.976	
**Q3**	1.446 (0.567, 3.686) 0.445	**2.314 (1.163, 4.605) 0.022**	1.309 (0.478, 3.584) 0.604		1.414 (0.547, 3.659) 0.480	**2.393 (1.174, 4.879) 0.022**	1.325 (0.487, 3.605) 0.585		1.285 (0.481, 3.436) 0.624	1.964 (0.892, 4.323) 0.113	1.246 (0.518, 2.999) 0.630	
**Q4**	1.098 (0.422, 2.855) 0.849	1.797 (0.654, 4.939) 0.264	**2.465 (1.041, 5.840) 0.048**		1.116 (0.427, 2.916) 0.824	1.870 (0.684, 5.112) 0.231	2.381 (1.012, 5.598) 0.055		0.876 (0.363, 2.114) 0.772	1.409 (0.439, 4.520) 0.572	1.895 (0.797, 4.504) 0.167	
**Walking difficulty**	**OR (95%CI) *P*-value**	**OR (95%CI) *P*-value**	**OR (95%CI) *P*-value**		**OR (95%CI) *P*-value**	**OR (95%CI) *P*-value**	**OR (95%CI) *P*-value**		**OR (95%CI) *P*-value**	**OR (95%CI) *P*-value**	**OR (95%CI) *P*-value**	
**Hydroxycotinine (Quartile)**				**0.020**				**0.026**				0.067
**Q1**	Reference	Reference	Reference		Reference	Reference	Reference		Reference	Reference	Reference	
**Q2**	2.542 (0.826, 7.824) 0.113	1.105 (0.426, 2.868) 0.838	0.843 (0.467, 1.522) 0.575		2.421 (0.784, 7.475) 0.134	1.156 (0.449, 2.975) 0.766	0.836 (0.475, 1.472) 0.539		2.358 (0.787, 7.061) 0.145	1.157 (0.484, 2.768) 0.747	1.004 (0.589, 1.712) 0.988	
**Q3**	1.119 (0.309, 4.043) 0.865	2.631 (0.987, 7.016) 0.062	2.631 (0.987, 7.016) 0.062		1.085 (0.299, 3.939) 0.902	2.761 (1.026, 7.431) 0.053	1.247 (0.562, 2.768) 0.591		1.020 (0.286, 3.638) 0.976	2.929 (1.063, 8.070) 0.054	1.242 (0.580, 2.658) 0.584	
**Q4**	1.254 (0.453,	2.114 (0.797,	**3.095 (1.552,**		1.281 (0.459,	2.235 (0.865,	**2.975 (1.497,**		1.140 (0.402,	2.027 (0.781,	**2.856 (1.465,**	

	3.469)<br>0.666	5.607)<br>0.142	**6.174)<br>0.003**		3.577)<br>0.640	5.771)<br>0.106	**5.913)<br>0.004**		3.234)<br>0.809	5.263)<br>0.166	**5.566)<br>0.007**	
**Difficulty dressing or bathing independently**	**OR (95%CI) *P*-value**	**OR (95%CI) *P*-value**	**OR (95%CI) *P*-value**		**OR (95%CI) *P*-value**	**OR (95%CI) *P*-value**	**OR (95%CI) *P*-value**		**OR (95%CI) *P*-value**	**OR (95%CI) *P*-value**	**OR (95%CI) *P*-value**	
**Hydroxycotinine (Quartile)**				**<0.001**				**<0.001**				**<0.001**
**Q1**	Reference	Reference	Reference		Reference	Reference	Reference		Reference	Reference	Reference	
**Q2**	0.476<br>(0.132,<br>1.722)<br>0.266	1.978<br>(0.806,<br>4.852)<br>0.146	1.131<br>(0.434,<br>2.946)<br>0.803		0.473<br>(0.132,<br>1.693)<br>0.258	1.990<br>(0.807,<br>4.907)<br>0.145	1.130<br>(0.434,<br>2.943)<br>0.804		0.478<br>(0.131,<br>1.745)<br>0.280	1.744<br>(0.663,<br>4.588)<br>0.276	1.175<br>(0.423,<br>3.264)<br>0.761	
**Q3**	0.000<br>(0.000,<br>0.000)<br><0.001	3.569<br>(0.932,<br>13.672)<br>0.072	1.423<br>(0.589,<br>3.438)<br>0.438		0.000<br>(0.000,<br>0.000)<br><0.001	3.589<br>(0.959,<br>13.438)<br>0.067	1.426<br>(0.591,<br>3.442)<br>0.435		0.000<br>(0.000,<br>0.000)<br><0.001	3.233<br>(0.774,<br>13.499)<br>0.127	1.370<br>(0.603,<br>3.113)<br>0.463	
**Q4**	0.481<br>(0.109,<br>2.120)<br>0.340	1.867<br>(0.590,<br>5.913)<br>0.296	1.100<br>(0.422,<br>2.866)<br>0.847		0.483<br>(0.109,<br>2.133)<br>0.344	1.880<br>(0.590,<br>5.993)<br>0.294	1.092<br>(0.412,<br>2.892)<br>0.860		0.457<br>(0.092,<br>2.265)<br>0.352	1.504<br>(0.468,<br>4.829)<br>0.503	0.899<br>(0.342,<br>2.363)<br>0.831	
**Difficulty running errands alone**	**OR (95%CI) *P*-value**	**OR (95%CI) *P*-value**	**OR (95%CI) *P*-value**		**OR (95%CI) *P*-value**	**OR (95%CI) *P*-value**	**OR (95%CI) *P*-value**		**OR (95%CI) *P*-value**	**OR (95%CI) *P*-value**	**OR (95%CI) *P*-value**	
**Hydroxycotinine (Quartile)**				0.644				0.615				0.494
**Q1**	Reference	Reference	Reference		Reference	Reference	Reference		Reference	Reference	Reference	
**Q2**	0.822<br>(0.207,	1.791<br>(0.763,	1.861<br>(0.902,		0.797<br>(0.200,	1.840<br>(0.775,	1.856<br>(0.890,		0.813<br>(0.198,	1.602<br>(0.739,	2.076<br>(0.989,	

	3.270) 0.783	4.203) 0.189	3.838) 0.102		3.181) 0.750	4.370) 0.176	3.871) 0.109		3.343) 0.778	3.473) 0.250	4.355) 0.071	
**Q3**	1.462 (0.330, 6.477) 0.620	**3.511 (1.392, 8.857) 0.011**	1.351 (0.578, 3.158) 0.492		1.435 (0.324, 6.355) 0.637	**3.601 (1.429, 9.075) 0.010**	1.364 (0.581, 3.204) 0.481		1.398 (0.292, 6.689) 0.680	2.842 (0.996, 8.109) 0.069	1.152 (0.464, 2.863) 0.764	
**Q4**	2.186 (0.561, 8.515) 0.268	**3.115 (1.188, 8.172) 0.027**	**2.598 (1.223, 5.517) 0.018**		2.216 (0.564, 8.705) 0.263	**3.213 (1.220, 8.465) 0.024**	**2.524 (1.178, 5.406) 0.023**		1.829 (0.438, 7.633) 0.420	2.276 (0.970, 5.345) 0.077	1.938 (0.840, 4.468) 0.140	

Model 1: no covariates were adjusted.

Model 2: gender was adjusted.

Model 3: gender, body mass index, race/ethnicity, data release cycle, education level, marital status, income to poverty ratio, alcohol use, hypertension, diabetes, cancer, and cardiovascular diseases were adjusted.

Supplementary Table 4-3. Association between serum cotinine level, cotinine, hydroxycotinine and adverse health outcomes, stratified by race/ethnicity, Weighted.

	**Model 1**				**Model 2**				**Model 3**			
	**Non-Hispanic White (n=693)**	**Non-Hispanic African American (n=650)**	**Others (n=827)**	***P*-interaction**	**Non-Hispanic White (n=693)**	**Non-Hispanic African American (n=650)**	**Others (n=827)**	***P*-interaction**	**Non-Hispanic White (n=693)**	**Non-Hispanic African American (n=650)**	**Others (n=827)**	***P*-interaction**
**Hearing difficulty**	**OR (95%CI) *P*-value**	**OR (95%CI) *P*-value**	**OR (95%CI) *P*-value**		**OR (95%CI) *P*-value**	**OR (95%CI) *P*-value**	**OR (95%CI) *P*-value**		**OR (95%CI) *P*-value**	**OR (95%CI) *P*-value**	**OR (95%CI) *P*-value**	
**Serum cotinine level**	0.836 (0.654, 1.067) 0.158	0.790 (0.569, 1.095) 0.165	0.702 (0.389, 1.269) 0.248	0.851	0.954 (0.787, 1.157) 0.637	0.927 (0.666, 1.291) 0.658	0.879 (0.591, 1.308) 0.530	0.929	0.937 (0.752, 1.167) 0.567	0.926 (0.653, 1.312) 0.669	0.860 (0.577, 1.281) 0.465	0.935
**Vision impairment**	**OR (95%CI) *P*-value**	**OR (95%CI) *P*-value**	**OR (95%CI) *P*-value**		**OR (95%CI) *P*-value**	**OR (95%CI) *P*-value**	**OR (95%CI) *P*-value**		**OR (95%CI) *P*-value**	**OR (95%CI) *P*-value**	**OR (95%CI) *P*-value**	

**Serum cotinine level**	0.861 (0.671, 1.104) 0.244	0.952 (0.778, 1.165) 0.635	0.990 (0.752, 1.303) 0.942	0.751	0.930 (0.740, 1.168) 0.538	1.019 (0.836, 1.242) 0.852	1.060 (0.821, 1.369) 0.656	0.758	0.785 (0.576, 1.068) 0.137	1.005 (0.786, 1.285) 0.969	1.054 (0.828, 1.342) 0.673	0.352
**Attention deficit hyperactivity disorder**	**OR (95%CI) *P*-value**	**OR (95%CI) *P*-value**	**OR (95%CI) *P*-value**		**OR (95%CI) *P*-value**	**OR (95%CI) *P*-value**	**OR (95%CI) *P*-value**		**OR (95%CI) *P*-value**	**OR (95%CI) *P*-value**	**OR (95%CI) *P*-value**	
**Serum cotinine level**	0.942 (0.855, 1.039) 0.241	1.092 (0.926, 1.288) 0.304	0.839 (0.656, 1.074) 0.171	0.179	0.983 (0.886, 1.090) 0.742	1.128 (0.963, 1.321) 0.144	0.880 (0.701, 1.104) 0.275	0.198	0.896 (0.789, 1.017) 0.104	1.089 (0.937, 1.265) 0.277	0.860 (0.670, 1.104) 0.249	0.146
**Walking difficulty**	**OR (95%CI) *P*-value**	**OR (95%CI) *P*-value**	**OR (95%CI) *P*-value**		**OR (95%CI) *P*-value**	**OR (95%CI) *P*-value**	**OR (95%CI) *P*-value**		**OR (95%CI) *P*-value**	**OR (95%CI) *P*-value**	**OR (95%CI) *P*-value**	
**Serum cotinine level**	1.025 (0.890, 1.181) 0.729	0.926 (0.805, 1.064) 0.283	**0.814 (0.668, 0.993) 0.049**	0.232	**1.213 (1.060, 1.388) 0.008**	1.079 (0.935, 1.245) 0.305	0.975 (0.838, 1.134) 0.741	0.137	1.170 (0.986, 1.389) 0.086	1.085 (0.922, 1.276) 0.338	0.986 (0.827, 1.176) 0.877	0.441
**Difficulty dressing or bathing independently**	**OR (95%CI) *P*-value**	**OR (95%CI) *P*-value**	**OR (95%CI) *P*-value**		**OR (95%CI) *P*-value**	**OR (95%CI) *P*-value**	**OR (95%CI) *P*-value**		**OR (95%CI) *P*-value**	**OR (95%CI) *P*-value**	**OR (95%CI) *P*-value**	
**Serum cotinine level**	0.922 (0.718, 1.185) 0.531	0.936 (0.715, 1.225) 0.631	0.934 (0.755, 1.155) 0.532	0.997	1.040 (0.821, 1.318) 0.746	1.059 (0.817, 1.373) 0.666	1.062 (0.878, 1.286) 0.538	0.990	0.993 (0.779, 1.266) 0.958	1.038 (0.776, 1.387) 0.804	1.070 (0.861, 1.329) 0.548	0.892
**Difficulty running errands alone**	**OR (95%CI) *P*-value**	**OR (95%CI) *P*-value**	**OR (95%CI) *P*-value**		**OR (95%CI) *P*-value**	**OR (95%CI) *P*-value**	**OR (95%CI) *P*-value**		**OR (95%CI) *P*-value**	**OR (95%CI) *P*-value**	**OR (95%CI) *P*-value**	

**Serum cotinine level**	1.057 (0.938, 1.190) 0.370	0.965 (0.771, 1.207) 0.755	0.811 (0.612, 1.076) 0.155	0.223	**1.166 (1.017, 1.337) 0.034**	1.058 (0.826, 1.354) 0.660	0.912 (0.722, 1.153) 0.447	0.174	1.079 (0.950, 1.226) 0.255	1.009 (0.777, 1.311) 0.945	0.886 (0.682, 1.153) 0.377	0.400
**Hearing difficulty**	**OR (95%CI) *P*-value**	**OR (95%CI) *P*-value**	**OR (95%CI) *P*-value**		**OR (95%CI) *P*-value**	**OR (95%CI) *P*-value**	**OR (95%CI) *P*-value**		**OR (95%CI) *P*-value**	**OR (95%CI) *P*-value**	**OR (95%CI) *P*-value**	
**Serum cotinine level (Quartile)**				0.915				0.916				0.933
**Q1**	Reference	Reference	Reference		Reference	Reference	Reference		Reference	Reference	Reference	
**Q2**	0.507 (0.216, 1.190) 0.128	0.512 (0.176, 1.494) 0.229	**0.273 (0.101, 0.735) 0.015**		0.601 (0.236, 1.529) 0.293	0.755 (0.242, 2.350) 0.630	**0.296 (0.108, 0.809) 0.024**		0.532 (0.202, 1.401) 0.219	0.795 (0.243, 2.598) 0.708	**0.304 (0.111, 0.835) 0.034**	
**Q3**	0.531 (0.236, 1.195) 0.135	0.393 (0.079, 1.954) 0.262	**0.314 (0.110, 0.894) 0.037**		0.587 (0.229, 1.499) 0.274	0.640 (0.125, 3.276) 0.596	0.468 (0.163, 1.345) 0.168		0.547 (0.204, 1.464) 0.246	0.506 (0.097, 2.647) 0.431	0.418 (0.148, 1.179) 0.118	
**Q4**	0.584 (0.267, 1.276) 0.186	0.516 (0.179, 1.491) 0.230	0.270 (0.062, 1.177) 0.090		0.953 (0.393, 2.316) 0.917	1.032 (0.297, 3.592) 0.960	0.529 (0.117, 2.395) 0.414		0.777 (0.347, 1.738) 0.547	0.956 (0.246, 3.720) 0.949	0.448 (0.098, 2.058) 0.316	
**Vision impairment**	**OR (95%CI) *P*-value**	**OR (95%CI) *P*-value**	**OR (95%CI) *P*-value**		**OR (95%CI) *P*-value**	**OR (95%CI) *P*-value**	**OR (95%CI) *P*-value**		**OR (95%CI) *P*-value**	**OR (95%CI) *P*-value**	**OR (95%CI) *P*-value**	
**Serum cotinine level (Quartile)**				0.886				0.892				0.977
**Q1**	Reference	Reference	Reference		Reference	Reference	Reference		Reference	Reference	Reference	
**Q2**	0.820 (0.285,	1.067 (0.315,	1.120 (0.370,		0.917 (0.309,	1.382 (0.400,	1.197 (0.386,		0.923 (0.302,	1.459 (0.403,	1.245 (0.385,	

		2.362) 0.716	3.618) 0.918	3.394) 0.843		2.716) 0.876	4.770) 0.613	3.714) 0.758		2.827) 0.890	5.279) 0.572	4.021) 0.719	
	**Q3**	1.329 (0.502, 3.522) 0.570	1.146 (0.440, 2.982) 0.782	1.309 (0.405, 4.234) 0.656		1.491 (0.540, 4.116) 0.446	1.559 (0.604, 4.028) 0.366	1.598 (0.499, 5.115) 0.436		1.471 (0.531, 4.074) 0.468	1.578 (0.575, 4.330) 0.388	1.426 (0.436, 4.662) 0.565	
	**Q4**	1.168 (0.416, 3.281) 0.769	0.803 (0.270, 2.390) 0.696	0.941 (0.303, 2.923) 0.917		1.573 (0.535, 4.626) 0.416	1.179 (0.399, 3.485) 0.768	1.305 (0.420, 4.053) 0.648		0.924 (0.270, 3.163) 0.902	0.932 (0.299, 2.906) 0.904	1.285 (0.397, 4.167) 0.681	
**Attention deficit hyperactivity disorder**		**OR (95%CI) *P*-value**	**OR (95%CI) *P*-value**	**OR (95%CI) *P*-value**		**OR (95%CI) *P*-value**	**OR (95%CI) *P*-value**	**OR (95%CI) *P*-value**		**OR (95%CI) *P*-value**	**OR (95%CI) *P*-value**	**OR (95%CI) *P*-value**	
**Serum cotinine level (Quartile)**					0.759				0.830				0.801
	**Q1**	Reference	Reference	Reference		Reference	Reference	Reference		Reference	Reference	Reference	
	**Q2**	0.769 (0.387, 1.532) 0.461	0.766 (0.263, 2.231) 0.628	1.515 (0.687, 3.342) 0.311		0.809 (0.407, 1.608) 0.550	0.891 (0.309, 2.573) 0.833	1.542 (0.698, 3.405) 0.292		0.790 (0.415, 1.507) 0.485	0.920 (0.302, 2.798) 0.885	1.451 (0.664, 3.170) 0.363	
	**Q3**	1.425 (0.659, 3.081) 0.374	0.983 (0.399, 2.422) 0.971	2.017 (0.917, 4.436) 0.090		1.535 (0.703, 3.348) 0.290	1.160 (0.467, 2.878) 0.751	2.157 (0.958, 4.855) 0.073		1.452 (0.666, 3.164) 0.362	0.998 (0.367, 2.720) 0.998	1.816 (0.808, 4.081) 0.167	
	**Q4**	1.409 (0.826, 2.402) 0.217	1.309 (0.532, 3.223) 0.562	1.408 (0.544, 3.642) 0.486		1.663 (0.977, 2.829) 0.070	1.611 (0.665, 3.900) 0.299	1.675 (0.659, 4.259) 0.286		1.121 (0.651, 1.932) 0.686	1.229 (0.448, 3.372) 0.694	1.447 (0.551, 3.800) 0.464	
**Walking difficulty**		**OR (95%CI) *P*-value**	**OR (95%CI) *P*-value**	**OR (95%CI) *P*-value**		**OR (95%CI) *P*-value**	**OR (95%CI) *P*-value**	**OR (95%CI) *P*-value**		**OR (95%CI) *P*-value**	**OR (95%CI) *P*-value**	**OR (95%CI) *P*-value**	

**Serum cotinine level (Quartile)**				0.596				0.564				0.492
**Q1**	Reference	Reference	Reference		Reference	Reference	Reference		Reference	Reference	Reference	
**Q2**	1.110 (0.581, 2.119) 0.754	0.501 (0.244, 1.029) 0.069	0.939 (0.488, 1.805) 0.850		1.404 (0.683, 2.887) 0.363	0.822 (0.366, 1.846) 0.638	1.056 (0.496, 2.248) 0.889		1.549 (0.778, 3.085) 0.230	0.767 (0.319, 1.841) 0.560	1.228 (0.569, 2.649) 0.608	
**Q3**	1.311 (0.623, 2.761) 0.480	0.774 (0.437, 1.370) 0.385	0.971 (0.488, 1.930) 0.934		1.713 (0.744, 3.943) 0.215	1.407 (0.724, 2.735) 0.321	1.373 (0.677, 2.781) 0.386		1.676 (0.692, 4.057) 0.268	1.245 (0.598, 2.593) 0.566	1.300 (0.633, 2.669) 0.485	
**Q4**	1.530 (0.757, 3.091) 0.245	0.598 (0.320, 1.116) 0.115	1.176 (0.533, 2.594) 0.691		**2.966 (1.538, 5.719) 0.003**	1.235 (0.629, 2.424) 0.544	**2.329 (1.070, 5.067) 0.041**		**2.396 (1.211, 4.741) 0.022**	0.972 (0.472, 2.001) 0.940	2.221 (0.924, 5.339) 0.092	
**Difficulty dressing or bathing independently**	**OR (95%CI) *P*-value**	**OR (95%CI) *P*-value**	**OR (95%CI) *P*-value**		**OR (95%CI) *P*-value**	**OR (95%CI) *P*-value**	**OR (95%CI) *P*-value**		**OR (95%CI) *P*-value**	**OR (95%CI) *P*-value**	**OR (95%CI) *P*-value**	
**Serum cotinine level (Quartile)**				0.888				0.712				0.681
**Q1**	Reference	Reference	Reference		Reference	Reference	Reference		Reference	Reference	Reference	
**Q2**	0.756 (0.239, 2.393) 0.637	0.807 (0.240, 2.719) 0.732	0.702 (0.253, 1.947) 0.501		0.902 (0.276, 2.951) 0.866	1.211 (0.363, 4.035) 0.758	0.787 (0.279, 2.217) 0.653		0.836 (0.250, 2.798) 0.775	1.188 (0.320, 4.417) 0.800	0.747 (0.274, 2.038) 0.576	
**Q3**	0.908 (0.213, 3.878) 0.897	1.273 (0.378, 4.285) 0.699	0.693 (0.259, 1.859) 0.472		1.063 (0.249, 4.538) 0.934	2.073 (0.635, 6.772) 0.236	0.959 (0.352, 2.610) 0.935		1.036 (0.233, 4.595) 0.964	1.812 (0.521, 6.301) 0.363	0.774 (0.281, 2.133) 0.626	

	**Q4**	0.651 (0.264, 1.605) 0.358	0.599 (0.148, 2.426) 0.477	1.089 (0.410, 2.896) 0.865		0.987 (0.432, 2.259) 0.976	1.087 (0.274, 4.307) 0.906	1.913 (0.675, 5.423) 0.231		0.815 (0.352, 1.888) 0.640	0.848 (0.213, 3.370) 0.817	1.592 (0.566, 4.476) 0.390	
**Difficulty running errands alone**		**OR (95%CI) *P*-value**	**OR (95%CI) *P*-value**	**OR (95%CI) *P*-value**		**OR (95%CI) *P*-value**	**OR (95%CI) *P*-value**	**OR (95%CI) *P*-value**		**OR (95%CI) *P*-value**	**OR (95%CI) *P*-value**	**OR (95%CI) *P*-value**	
**Serum cotinine level (Quartile)**					0.066				**0.044**				0.113
	**Q1**	Reference	Reference	Reference		Reference	Reference	Reference		Reference	Reference	Reference	
	**Q2**	**0.377 (0.184, 0.773) 0.012**	0.911 (0.376, 2.209) 0.839	1.341 (0.556, 3.232) 0.518		**0.427 (0.202, 0.899) 0.032**	1.292 (0.544, 3.069) 0.566	1.455 (0.613, 3.457) 0.401		**0.388 (0.168, 0.893) 0.040**	1.275 (0.528, 3.078) 0.596	1.243 (0.470, 3.286) 0.666	
	**Q3**	1.354 (0.555, 3.306) 0.510	0.978 (0.316, 3.026) 0.969	1.152 (0.517, 2.566) 0.731		1.585 (0.631, 3.983) 0.334	1.453 (0.476, 4.436) 0.517	1.450 (0.680, 3.093) 0.344		1.384 (0.510, 3.758) 0.532	1.223 (0.400, 3.741) 0.728	1.069 (0.501, 2.282) 0.866	
	**Q4**	1.706 (0.809, 3.594) 0.169	0.978 (0.362, 2.640) 0.966	1.512 (0.503, 4.547) 0.467		**2.547 (1.261, 5.142) 0.014**	1.611 (0.561, 4.626) 0.382	2.327 (0.771, 7.024) 0.144		1.763 (0.796, 3.904) 0.180	1.182 (0.426, 3.280) 0.752	1.786 (0.609, 5.239) 0.305	
**Hearing difficulty**		**OR (95%CI) *P*-value**	**OR (95%CI) *P*-value**	**OR (95%CI) *P*-value**		**OR (95%CI) *P*-value**	**OR (95%CI) *P*-value**	**OR (95%CI) *P*-value**		**OR (95%CI) *P*-value**	**OR (95%CI) *P*-value**	**OR (95%CI) *P*-value**	
**Cotinine**		0.687 (0.457, 1.034) 0.079	0.747 (0.504, 1.108) 0.155	0.604 (0.244, 1.491) 0.280	0.886	0.906 (0.659, 1.248) 0.551	0.949 (0.650, 1.385) 0.788	0.843 (0.497, 1.427) 0.528	0.932	0.884 (0.610, 1.281) 0.521	0.958 (0.642, 1.430) 0.837	0.822 (0.489, 1.381) 0.466	0.888

**Vision impairment**	**OR (95%CI) *P*-value**	**OR (95%CI) *P*-value**	**OR (95%CI) *P*-value**		**OR (95%CI) *P*-value**	**OR (95%CI) *P*-value**	**OR (95%CI) *P*-value**		**OR (95%CI) *P*-value**	**OR (95%CI) *P*-value**	**OR (95%CI) *P*-value**	
**Cotinine**	0.760 (0.471, 1.228) 0.269	0.930 (0.713, 1.212) 0.594	0.977 (0.694, 1.374) 0.893	0.713	0.878 (0.577, 1.335) 0.546	1.021 (0.791, 1.319) 0.872	1.070 (0.786, 1.456) 0.671	0.775	0.666 (0.366, 1.213) 0.197	1.009 (0.743, 1.370) 0.956	1.061 (0.790, 1.425) 0.700	0.421
**Attention deficit hyperactivity disorder**	**OR (95%CI) *P*-value**	**OR (95%CI) *P*-value**	**OR (95%CI) *P*-value**		**OR (95%CI) *P*-value**	**OR (95%CI) *P*-value**	**OR (95%CI) *P*-value**		**OR (95%CI) *P*-value**	**OR (95%CI) *P*-value**	**OR (95%CI) *P*-value**	
**Cotinine**	0.909 (0.786, 1.051) 0.204	1.107 (0.885, 1.386) 0.378	0.799 (0.584, 1.091) 0.166	0.235	0.971 (0.831, 1.134) 0.712	1.155 (0.936, 1.424) 0.186	0.851 (0.642, 1.128) 0.269	0.258	0.845 (0.691, 1.032) 0.113	1.104 (0.907, 1.346) 0.334	0.824 (0.604, 1.124) 0.233	0.205
**Walking difficulty**	**OR (95%CI) *P*-value**	**OR (95%CI) *P*-value**	**OR (95%CI) *P*-value**		**OR (95%CI) *P*-value**	**OR (95%CI) *P*-value**	**OR (95%CI) *P*-value**		**OR (95%CI) *P*-value**	**OR (95%CI) *P*-value**	**OR (95%CI) *P*-value**	
**Cotinine**	1.012 (0.810, 1.265) 0.918	0.852 (0.717, 1.012) 0.076	0.742 (0.554, 0.995) 0.053	0.282	**1.330 (1.084, 1.633) 0.010**	1.054 (0.893, 1.244) 0.540	0.953 (0.778, 1.167) 0.644	0.068	1.252 (0.966, 1.622) 0.103	1.059 (0.881, 1.273) 0.548	0.968 (0.767, 1.221) 0.784	0.382
**Difficulty dressing or bathing independently**	**OR (95%CI) *P*-value**	**OR (95%CI) *P*-value**	**OR (95%CI) *P*-value**		**OR (95%CI) *P*-value**	**OR (95%CI) *P*-value**	**OR (95%CI) *P*-value**		**OR (95%CI) *P*-value**	**OR (95%CI) *P*-value**	**OR (95%CI) *P*-value**	
**Cotinine**	0.874 (0.599, 1.274) 0.487	0.871 (0.611, 1.243) 0.451	0.907 (0.676, 1.216) 0.517	0.974	1.088 (0.782, 1.513) 0.620	1.040 (0.754, 1.433) 0.813	1.075 (0.838, 1.379) 0.572	0.973	1.016 (0.721, 1.433) 0.928	1.006 (0.705, 1.434) 0.975	1.081 (0.820, 1.425) 0.587	0.909

**Difficulty running errands alone**	**OR (95%CI) *P*-value**	**OR (95%CI) *P*-value**	**OR (95%CI) *P*-value**		**OR (95%CI) *P*-value**	**OR (95%CI) *P*-value**	**OR (95%CI) *P*-value**		**OR (95%CI) *P*-value**	**OR (95%CI) *P*-value**	**OR (95%CI) *P*-value**	
**Cotinine**	1.072 (0.897, 1.282) 0.447	0.896 (0.708, 1.134) 0.365	0.758 (0.508, 1.132) 0.183	0.198	**1.259 (1.036, 1.530) 0.026**	1.019 (0.790, 1.313) 0.887	0.892 (0.654, 1.218) 0.477	0.094	1.118 (0.928, 1.348) 0.253	0.954 (0.730, 1.248) 0.735	0.859 (0.608, 1.214) 0.399	0.302
**Hearing difficulty**	**OR (95%CI) *P*-value**	**OR (95%CI) *P*-value**	**OR (95%CI) *P*-value**		**OR (95%CI) *P*-value**	**OR (95%CI) *P*-value**	**OR (95%CI) *P*-value**		**OR (95%CI) *P*-value**	**OR (95%CI) *P*-value**	**OR (95%CI) *P*-value**	
**Cotinine (Quartile)**				0.853				0.944				0.930
**Q1**	Reference	Reference	Reference		Reference	Reference	Reference		Reference	Reference	Reference	
**Q2**	**0.290 (0.138, 0.610) 0.003**	0.514 (0.181, 1.462) 0.221	**0.227 (0.085, 0.606) 0.006**		**0.366 (0.149, 0.899) 0.036**	0.674 (0.224, 2.026) 0.488	**0.263 (0.095, 0.730) 0.015**		**0.286 (0.110, 0.740) 0.020**	0.586 (0.178, 1.928) 0.392	**0.265 (0.097, 0.720) 0.019**	
**Q3**	0.410 (0.171, 0.985) 0.054	0.349 (0.068, 1.794) 0.216	**0.270 (0.093, 0.781) 0.021**		0.490 (0.185, 1.292) 0.159	0.575 (0.111, 2.968) 0.513	0.445 (0.158, 1.253) 0.135		0.490 (0.168, 1.428) 0.208	0.455 (0.083, 2.480) 0.375	0.403 (0.151, 1.076) 0.087	
**Q4**	**0.438 (0.221, 0.866) 0.024**	0.469 (0.162, 1.359) 0.172	0.231 (0.053, 1.008) 0.060		0.839 (0.381, 1.848) 0.666	1.074 (0.305, 3.784) 0.912	0.514 (0.118, 2.236) 0.381		0.629 (0.309, 1.278) 0.217	0.951 (0.244, 3.706) 0.943	0.442 (0.102, 1.914) 0.290	
**Vision impairment**	**OR (95%CI) *P*-value**	**OR (95%CI) *P*-value**	**OR (95%CI) *P*-value**		**OR (95%CI) *P*-value**	**OR (95%CI) *P*-value**	**OR (95%CI) *P*-value**		**OR (95%CI) *P*-value**	**OR (95%CI) *P*-value**	**OR (95%CI) *P*-value**	
**Cotinine (Quartile)**				0.504				0.665				0.308
**Q1**	Reference	Reference	Reference		Reference	Reference	Reference		Reference	Reference	Reference	

**Q2**	0.579 (0.188, 1.785) 0.348	0.942 (0.296, 3.002) 0.921	1.010 (0.413, 2.468) 0.983		0.706 (0.221, 2.259) 0.562	1.119 (0.345, 3.632) 0.853	1.119 (0.455, 2.754) 0.808		0.693 (0.216, 2.216) 0.544	1.152 (0.354, 3.748) 0.817	1.111 (0.442, 2.795) 0.825	
**Q3**	1.416 (0.545, 3.674) 0.480	0.979 (0.391, 2.449) 0.964	1.189 (0.322, 4.389) 0.796		1.678 (0.623, 4.521) 0.314	1.359 (0.533, 3.464) 0.525	1.531 (0.423, 5.540) 0.520		1.768 (0.649, 4.819) 0.280	1.456 (0.493, 4.296) 0.506	1.334 (0.354, 5.035) 0.676	
**Q4**	0.742 (0.254, 2.167) 0.589	0.642 (0.241, 1.715) 0.383	1.315 (0.412, 4.195) 0.647		1.093 (0.357, 3.347) 0.877	0.974 (0.363, 2.613) 0.959	1.958 (0.609, 6.293) 0.268		0.581 (0.158, 2.127) 0.423	0.797 (0.278, 2.289) 0.679	1.981 (0.621, 6.321) 0.264	
**Attention deficit hyperactivity disorder**	**OR (95%CI) *P*-value**	**OR (95%CI) *P*-value**	**OR (95%CI) *P*-value**		**OR (95%CI) *P*-value**	**OR (95%CI) *P*-value**	**OR (95%CI) *P*-value**		**OR (95%CI) *P*-value**	**OR (95%CI) *P*-value**	**OR (95%CI) *P*-value**	
**Cotinine (Quartile)**				0.485				0.648				0.449
**Q1**	Reference	Reference	Reference		Reference	Reference	Reference		Reference	Reference	Reference	
**Q2**	0.864 (0.400, 1.865) 0.712	0.907 (0.362, 2.271) 0.836	1.558 (0.704, 3.446) 0.281		0.984 (0.441, 2.194) 0.969	1.003 (0.414, 2.434) 0.994	1.579 (0.715, 3.487) 0.267		0.971 (0.438, 2.151) 0.942	1.015 (0.378, 2.723) 0.977	1.433 (0.630, 3.262) 0.403	
**Q3**	1.519 (0.670, 3.444) 0.324	0.585 (0.226, 1.513) 0.276	1.862 (0.859, 4.037) 0.125		1.694 (0.735, 3.904) 0.225	0.707 (0.270, 1.853) 0.485	2.066 (0.934, 4.574) 0.083		1.678 (0.697, 4.041) 0.264	0.637 (0.210, 1.934) 0.438	1.736 (0.780, 3.860) 0.194	
**Q4**	1.395 (0.736, 2.644) 0.315	1.147 (0.484, 2.720) 0.757	1.669 (0.664, 4.198) 0.284		1.758 (0.896, 3.449) 0.111	1.433 (0.605, 3.394) 0.420	2.061 (0.825, 5.148) 0.131		1.145 (0.550, 2.385) 0.722	1.120 (0.403, 3.116) 0.830	1.695 (0.636, 4.520) 0.306	

**Walking difficulty**	**OR (95%CI) *P*-value**	**OR (95%CI) *P*-value**	**OR (95%CI) *P*-value**		**OR (95%CI) *P*-value**	**OR (95%CI) *P*-value**	**OR (95%CI) *P*-value**		**OR (95%CI) *P*-value**	**OR (95%CI) *P*-value**	**OR (95%CI) *P*-value**	
**Cotinine (Quartile)**				0.284				0.280				0.490
**Q1**	Reference	Reference	Reference		Reference	Reference	Reference		Reference	Reference	Reference	
**Q2**	0.747 (0.366, 1.524) 0.428	0.621 (0.293, 1.315) 0.222	0.776 (0.412, 1.464) 0.440		1.113 (0.475, 2.612) 0.807	0.841 (0.377, 1.877) 0.675	0.903 (0.449, 1.817) 0.777		1.076 (0.445, 2.603) 0.873	0.778 (0.344, 1.761) 0.555	1.014 (0.502, 2.049) 0.969	
**Q3**	1.387 (0.627, 3.068) 0.425	**0.546 (0.313, 0.951) 0.040**	0.661 (0.339, 1.289) 0.233		2.043 (0.869, 4.803) 0.111	1.007 (0.544, 1.866) 0.982	1.029 (0.522, 2.027) 0.935		2.159 (0.900, 5.182) 0.103	0.945 (0.480, 1.859) 0.872	0.940 (0.474, 1.861) 0.860	
**Q4**	1.165 (0.598, 2.267) 0.657	**0.489 (0.284, 0.840) 0.014**	0.840 (0.369, 1.910) 0.680		**2.704 (1.484, 4.925) 0.003**	1.056 (0.571, 1.952) 0.864	1.859 (0.868, 3.984) 0.120		1.884 (0.909, 3.904) 0.107	0.879 (0.465, 1.662) 0.696	1.780 (0.746, 4.246) 0.211	
**Difficulty dressing or bathing independently**	**OR (95%CI) *P*-value**	**OR (95%CI) *P*-value**	**OR (95%CI) *P*-value**		**OR (95%CI) *P*-value**	**OR (95%CI) *P*-value**	**OR (95%CI) *P*-value**		**OR (95%CI) *P*-value**	**OR (95%CI) *P*-value**	**OR (95%CI) *P*-value**	
**Cotinine (Quartile)**				0.942				0.874				0.804
**Q1**	Reference	Reference	Reference		Reference	Reference	Reference		Reference	Reference	Reference	
**Q2**	0.952 (0.268, 3.382) 0.940	0.582 (0.138, 2.453) 0.466	0.640 (0.240, 1.708) 0.379		1.322 (0.363, 4.811) 0.675	0.756 (0.176, 3.241) 0.709	0.760 (0.280, 2.060) 0.593		1.165 (0.312, 4.353) 0.823	0.711 (0.148, 3.412) 0.675	0.724 (0.278, 1.889) 0.518	
**Q3**	1.003 (0.230,	0.796 (0.211,	0.653 (0.247,		1.286 (0.303,	1.339 (0.374,	0.999 (0.377,		1.344 (0.329,	1.234 (0.320,	0.805 (0.289,	

		4.376) 0.997	3.000) 0.738	1.727) 0.397		5.447) 0.735	4.790) 0.657	2.650) 0.999		5.496) 0.686	4.752) 0.764	2.240) 0.683	
	**Q4**	0.751 (0.276, 2.042) 0.578	0.416 (0.102, 1.687) 0.228	0.967 (0.370, 2.527) 0.946		1.372 (0.581, 3.240) 0.476	0.814 (0.198, 3.346) 0.778	1.921 (0.678, 5.448) 0.228		1.015 (0.434, 2.374) 0.973	0.644 (0.151, 2.747) 0.560	1.643 (0.592, 4.562) 0.354	
**Difficulty running errands alone**		**OR (95%CI) *P*-value**	**OR (95%CI) *P*-value**	**OR (95%CI) *P*-value**		**OR (95%CI) *P*-value**	**OR (95%CI) *P*-value**	**OR (95%CI) *P*-value**		**OR (95%CI) *P*-value**	**OR (95%CI) *P*-value**	**OR (95%CI) *P*-value**	
**Cotinine (Quartile)**					0.452				0.676				0.745
	**Q1**	Reference	Reference	Reference		Reference	Reference	Reference		Reference	Reference	Reference	
	**Q2**	0.672 (0.282, 1.600) 0.376	0.796 (0.282, 2.253) 0.670	1.015 (0.475, 2.171) 0.970		0.879 (0.360, 2.145) 0.778	0.998 (0.361, 2.762) 0.998	1.140 (0.552, 2.353) 0.726		0.792 (0.313, 2.004) 0.630	0.929 (0.332, 2.599) 0.890	0.916 (0.410, 2.045) 0.832	
	**Q3**	1.451 (0.574, 3.668) 0.436	0.550 (0.156, 1.930) 0.357	1.082 (0.546, 2.145) 0.822		1.824 (0.706, 4.716) 0.224	0.841 (0.257, 2.758) 0.777	1.486 (0.782, 2.825) 0.235		1.733 (0.620, 4.846) 0.309	0.715 (0.219, 2.337) 0.586	1.107 (0.568, 2.157) 0.768	
	**Q4**	1.870 (0.894, 3.915) 0.106	0.795 (0.285, 2.215) 0.664	0.958 (0.305, 3.003) 0.942		**3.235 (1.666, 6.282) 0.002**	1.375 (0.463, 4.089) 0.570	1.609 (0.511, 5.068) 0.422		2.092 (0.989, 4.425) 0.070	1.025 (0.344, 3.052) 0.966	1.160 (0.380, 3.540) 0.798	
**Hearing difficulty**		**OR (95%CI) *P*-value**	**OR (95%CI) *P*-value**	**OR (95%CI) *P*-value**		**OR (95%CI) *P*-value**	**OR (95%CI) *P*-value**	**OR (95%CI) *P*-value**		**OR (95%CI) *P*-value**	**OR (95%CI) *P*-value**	**OR (95%CI) *P*-value**	
**Hydroxycotinine**		0.800 (0.436, 1.470) 0.477	0.372 (0.064, 2.168) 0.278	0.356 (0.071, 1.794) 0.218	0.473	0.944 (0.594, 1.498) 0.807	0.507 (0.084, 3.058) 0.463	0.704 (0.203, 2.447) 0.584	0.72	0.895 (0.536, 1.493) 0.674	0.457 (0.073, 2.850) 0.410	0.632 (0.154, 2.600) 0.531	0.672

**Vision impairment**	**OR (95%CI) *P*-value**	**OR (95%CI) *P*-value**	**OR (95%CI) *P*-value**		**OR (95%CI) *P*-value**	**OR (95%CI) *P*-value**	**OR (95%CI) *P*-value**		**OR (95%CI) *P*-value**	**OR (95%CI) *P*-value**	**OR (95%CI) *P*-value**	
**Hydroxycotinine**	0.775 (0.440, 1.364) 0.382	0.914 (0.481, 1.739) 0.786	1.040 (0.391, 2.764) 0.938	0.866	0.880 (0.505, 1.532) 0.653	1.083 (0.574, 2.044) 0.806	1.255 (0.525, 2.998) 0.613	0.787	0.587 (0.281, 1.224) 0.169	0.993 (0.398, 2.477) 0.988	1.257 (0.536, 2.951) 0.604	0.428
**Attention deficit hyperactivity disorder**	**OR (95%CI) *P*-value**	**OR (95%CI) *P*-value**	**OR (95%CI) *P*-value**		**OR (95%CI) *P*-value**	**OR (95%CI) *P*-value**	**OR (95%CI) *P*-value**		**OR (95%CI) *P*-value**	**OR (95%CI) *P*-value**	**OR (95%CI) *P*-value**	
**Hydroxycotinine**	0.880 (0.650, 1.192) 0.414	1.404 (0.791, 2.491) 0.253	0.521 (0.175, 1.552) 0.249	0.205	0.966 (0.701, 1.331) 0.834	1.544 (0.870, 2.742) 0.146	0.613 (0.223, 1.684) 0.348	0.218	0.767 (0.530, 1.111) 0.174	1.368 (0.764, 2.453) 0.303	0.573 (0.184, 1.786) 0.347	0.224
**Walking difficulty**	**OR (95%CI) *P*-value**	**OR (95%CI) *P*-value**	**OR (95%CI) *P*-value**		**OR (95%CI) *P*-value**	**OR (95%CI) *P*-value**	**OR (95%CI) *P*-value**		**OR (95%CI) *P*-value**	**OR (95%CI) *P*-value**	**OR (95%CI) *P*-value**	
**Hydroxycotinine**	1.144 (0.808, 1.619) 0.454	1.097 (0.694, 1.734) 0.693	0.595 (0.325, 1.090) 0.101	0.208	**1.600 (1.151, 2.224) 0.008**	1.630 (1.005, 2.646) 0.055	1.033 (0.673, 1.585) 0.884	0.268	1.493 (0.981, 2.272) 0.074	1.693 (0.936, 3.063) 0.095	1.075 (0.621, 1.858) 0.799	0.510
**Difficulty dressing or bathing independently**	**OR (95%CI) *P*-value**	**OR (95%CI) *P*-value**	**OR (95%CI) *P*-value**		**OR (95%CI) *P*-value**	**OR (95%CI) *P*-value**	**OR (95%CI) *P*-value**		**OR (95%CI) *P*-value**	**OR (95%CI) *P*-value**	**OR (95%CI) *P*-value**	
**Hydroxycotinine**	0.851 (0.437, 1.660) 0.639	1.085 (0.471, 2.499) 0.848	0.851 (0.451, 1.608) 0.622	0.860	1.025 (0.523, 2.008) 0.942	1.453 (0.609, 3.468) 0.405	1.227 (0.731, 2.060) 0.444	0.826	0.915 (0.462, 1.813) 0.802	1.440 (0.499, 4.157) 0.507	1.301 (0.690, 2.453) 0.425	0.711

**Difficulty running errands alone**	**OR (95%CI) *P*-value**	**OR (95%CI) *P*-value**	**OR (95%CI) *P*-value**		**OR (95%CI) *P*-value**	**OR (95%CI) *P*-value**	**OR (95%CI) *P*-value**		**OR (95%CI) *P*-value**	**OR (95%CI) *P*-value**	**OR (95%CI) *P*-value**	
**Hydroxycotinine**	1.181 (0.857, 1.629) 0.315	1.247 (0.514, 3.025) 0.628	0.485 (0.187, 1.255) 0.144	0.214	1.435 (0.981, 2.099) 0.071	1.570 (0.596, 4.137) 0.367	0.710 (0.314, 1.604) 0.415	0.253	1.204 (0.838, 1.730) 0.326	1.417 (0.488, 4.116) 0.528	0.633 (0.232, 1.730) 0.382	0.451
**Hearing difficulty**	**OR (95%CI) *P*-value**	**OR (95%CI) *P*-value**	**OR (95%CI) *P*-value**		**OR (95%CI) *P*-value**	**OR (95%CI) *P*-value**	**OR (95%CI) *P*-value**		**OR (95%CI) *P*-value**	**OR (95%CI) *P*-value**	**OR (95%CI) *P*-value**	
**Hydroxycotinine (Quartile)**				0.854				0.941				0.974
**Q1**	Reference	Reference	Reference		Reference	Reference	Reference		Reference	Reference	Reference	
**Q2**	1.087 (0.510, 2.314) 0.831	1.097 (0.218, 5.518) 0.911	1.462 (0.500, 4.272) 0.492		0.952 (0.414, 2.190) 0.909	1.144 (0.206, 6.337) 0.879	1.085 (0.346, 3.396) 0.890		1.016 (0.456, 2.263) 0.969	1.092 (0.182, 6.569) 0.925	1.221 (0.404, 3.693) 0.728	
**Q3**	0.787 (0.421, 1.474) 0.460	1.059 (0.315, 3.562) 0.927	0.504 (0.169, 1.502) 0.227		0.711 (0.331, 1.525) 0.388	1.138 (0.307, 4.214) 0.848	0.505 (0.167, 1.527) 0.235		0.672 (0.311, 1.449) 0.324	0.966 (0.231, 4.045) 0.963	0.501 (0.176, 1.423) 0.212	
**Q4**	0.922 (0.437, 1.946) 0.832	0.809 (0.194, 3.379) 0.774	0.869 (0.293, 2.575) 0.801		1.073 (0.456, 2.520) 0.873	1.056 (0.239, 4.661) 0.943	1.080 (0.340, 3.432) 0.896		0.991 (0.428, 2.295) 0.984	0.940 (0.189, 4.669) 0.941	0.937 (0.290, 3.023) 0.914	
**Vision impairment**	**OR (95%CI) *P*-value**	**OR (95%CI) *P*-value**	**OR (95%CI) *P*-value**		**OR (95%CI) *P*-value**	**OR (95%CI) *P*-value**	**OR (95%CI) *P*-value**		**OR (95%CI) *P*-value**	**OR (95%CI) *P*-value**	**OR (95%CI) *P*-value**	
**Hydroxycotinine (Quartile)**				0.886				0.865				0.994

**Q1**	Reference	Reference	Reference		Reference	Reference	Reference		Reference	Reference	Reference	
**Q2**	1.117 (0.333, 3.747) 0.859	1.279 (0.470, 3.479) 0.633	0.760 (0.322, 1.792) 0.535		1.054 (0.318, 3.493) 0.932	1.292 (0.471, 3.550) 0.622	0.670 (0.284, 1.578) 0.366		1.091 (0.342, 3.480) 0.885	1.065 (0.383, 2.965) 0.905	0.807 (0.321, 2.028) 0.655	
**Q3**	1.394 (0.485, 4.001) 0.541	1.055 (0.429, 2.599) 0.907	1.214 (0.656, 2.244) 0.541		1.443 (0.483, 4.310) 0.516	1.123 (0.455, 2.769) 0.803	1.226 (0.676, 2.221) 0.507		1.426 (0.501, 4.059) 0.515	0.922 (0.383, 2.221) 0.859	1.165 (0.643, 2.110) 0.620	
**Q4**	1.583 (0.525, 4.771) 0.420	1.010 (0.411, 2.484) 0.983	0.548 (0.180, 1.668) 0.297		1.783 (0.559, 5.684) 0.336	1.170 (0.483, 2.834) 0.731	0.610 (0.195, 1.903) 0.400		1.157 (0.316, 4.227) 0.828	0.786 (0.334, 1.848) 0.588	0.596 (0.183, 1.940) 0.402	
**Attention deficit hyperactivity disorder Hydroxycotinine (Quartile)**	**OR (95%CI) *P*-value**	**OR (95%CI) *P*-value**	**OR (95%CI) *P*-value**	0.888	**OR (95%CI) *P*-value**	**OR (95%CI) *P*-value**	**OR (95%CI) *P*-value**	0.893	**OR (95%CI) *P*-value**	**OR (95%CI) *P*-value**	**OR (95%CI) *P*-value**	0.969
**Q1**	Reference	Reference	Reference		Reference	Reference	Reference		Reference	Reference	Reference	
**Q2**	1.503 (0.689, 3.277) 0.313	1.181 (0.372, 3.750) 0.779	1.064 (0.520, 2.180) 0.866		1.484 (0.687, 3.209) 0.323	1.153 (0.365, 3.647) 0.810	0.999 (0.487, 2.053) 0.999		1.443 (0.625, 3.331) 0.402	0.947 (0.275, 3.260) 0.932	1.054 (0.491, 2.261) 0.894	
**Q3**	1.667 (0.794, 3.503) 0.186	1.732 (0.580, 5.169) 0.332	1.574 (0.706, 3.510) 0.276		1.734 (0.830, 3.624) 0.153	1.783 (0.600, 5.300) 0.306	1.540 (0.682, 3.480) 0.307		1.533 (0.735, 3.196) 0.270	1.416 (0.443, 4.527) 0.565	1.298 (0.565, 2.980) 0.547	
**Q4**	**1.893 (1.074, 3.337) 0.034**	1.431 (0.452, 4.527) 0.546	0.902 (0.339, 2.396) 0.837		**2.034 (1.178, 3.512) 0.016**	1.514 (0.490, 4.676) 0.476	0.984 (0.382, 2.535) 0.974		1.394 (0.791, 2.456) 0.267	1.031 (0.308, 3.455) 0.961	0.859 (0.306, 2.407) 0.776	

**Walking difficulty**	**OR (95%CI) *P*-value**	**OR (95%CI) *P*-value**	**OR (95%CI) *P*-value**		**OR (95%CI) *P*-value**	**OR (95%CI) *P*-value**	**OR (95%CI) *P*-value**		**OR (95%CI) *P*-value**	**OR (95%CI) *P*-value**	**OR (95%CI) *P*-value**	
**Hydroxycotinine (Quartile)**				0.817				0.812				0.674
**Q1**	Reference	Reference	Reference		Reference	Reference	Reference		Reference	Reference	Reference	
**Q2**	1.235 (0.602, 2.534) 0.568	1.104 (0.501, 2.433) 0.808	1.664 (0.767, 3.606) 0.206		1.137 (0.576, 2.246) 0.714	1.101 (0.503, 2.408) 0.812	1.304 (0.614, 2.773) 0.494		1.192 (0.608, 2.339) 0.615	0.899 (0.362, 2.233) 0.821	1.754 (0.770, 3.998) 0.199	
**Q3**	1.370 (0.692, 2.714) 0.373	1.465 (0.656, 3.274) 0.358	**2.182 (1.071, 4.442) 0.039**		1.521 (0.745, 3.108) 0.258	1.687 (0.708, 4.020) 0.246	**2.232 (1.126, 4.425) 0.028**		1.477 (0.701, 3.110) 0.319	1.436 (0.575, 3.588) 0.449	2.272 (1.013, 5.097) 0.063	
**Q4**	1.860 (0.971, 3.566) 0.070	1.153 (0.537, 2.474) 0.718	1.818 (0.764, 4.330) 0.186		**2.493 (1.386, 4.486) 0.005**	1.516 (0.712, 3.227) 0.289	2.342 (0.985, 5.567) 0.063		**2.232 (1.195, 4.170) 0.022**	1.135 (0.492, 2.618) 0.769	2.309 (0.867, 6.152) 0.112	
**Difficulty dressing or bathing independently**	**OR (95%CI) *P*-value**	**OR (95%CI) *P*-value**	**OR (95%CI) *P*-value**		**OR (95%CI) *P*-value**	**OR (95%CI) *P*-value**	**OR (95%CI) *P*-value**		**OR (95%CI) *P*-value**	**OR (95%CI) *P*-value**	**OR (95%CI) *P*-value**	
**Hydroxycotinine (Quartile)**				0.143				0.084				0.054
**Q1**	Reference	Reference	Reference		Reference	Reference	Reference		Reference	Reference	Reference	
**Q2**	1.390 (0.465, 4.156) 0.559	1.189 (0.334, 4.237) 0.791	1.058 (0.449, 2.493) 0.898		1.278 (0.399, 4.096) 0.682	1.207 (0.339, 4.295) 0.773	0.838 (0.362, 1.939) 0.682		1.130 (0.302, 4.230) 0.858	1.025 (0.284, 3.698) 0.970	0.945 (0.374, 2.386) 0.906	

Q3	1.487 (0.452, 4.889) 0.518	1.806 (0.559, 5.834) 0.330	0.665 (0.227, 1.944) 0.461		1.532 (0.451, 5.208) 0.500	1.997 (0.586, 6.803) 0.277	0.659 (0.226, 1.919) 0.450		1.402 (0.401, 4.897) 0.603	1.632 (0.515, 5.177) 0.417	0.541 (0.177, 1.651) 0.296	
Q4	0.740 (0.292, 1.878) 0.531	0.788 (0.209, 2.966) 0.727	1.562 (0.553, 4.412) 0.406		0.831 (0.326, 2.118) 0.701	0.976 (0.264, 3.613) 0.971	1.879 (0.646, 5.469) 0.256		0.671 (0.216, 2.089) 0.501	0.711 (0.203, 2.493) 0.601	1.687 (0.568, 5.011) 0.360	
**Difficulty running errands alone**	**OR (95%CI) *P*-value**	**OR (95%CI) *P*-value**	**OR (95%CI) *P*-value**		**OR (95%CI) *P*-value**	**OR (95%CI) *P*-value**	**OR (95%CI) *P*-value**		**OR (95%CI) *P*-value**	**OR (95%CI) *P*-value**	**OR (95%CI) *P*-value**	
**Hydroxycotinine (Quartile)**				0.776				0.792				0.692
**Q1**	Reference	Reference	Reference		Reference	Reference	Reference		Reference	Reference	Reference	
**Q2**	1.579 (0.664, 3.754) 0.309	1.881 (0.785, 4.506) 0.166	2.261 (0.929, 5.505) 0.081		1.491 (0.612, 3.633) 0.386	1.895 (0.811, 4.424) 0.150	1.956 (0.817, 4.684) 0.142		1.456 (0.618, 3.430) 0.402	1.501 (0.594, 3.792) 0.403	2.094 (0.838, 5.231) 0.132	
**Q3**	1.699 (0.648, 4.454) 0.289	1.972 (0.742, 5.242) 0.182	1.979 (0.765, 5.114) 0.168		1.798 (0.667, 4.845) 0.254	2.144 (0.790, 5.819) 0.144	1.974 (0.760, 5.130) 0.172		1.567 (0.538, 4.563) 0.422	1.658 (0.617, 4.450) 0.330	1.537 (0.557, 4.241) 0.418	
**Q4**	**2.928 (1.279, 6.704) 0.016**	1.275 (0.434, 3.741) 0.661	2.100 (0.783, 5.634) 0.150		**3.465 (1.511, 7.945) 0.006**	1.514 (0.521, 4.398) 0.451	2.456 (0.915, 6.588) 0.084		2.574 (1.058, 6.263) 0.053	1.028 (0.359, 2.941) 0.960	2.023 (0.728, 5.621) 0.194	

Model 1: no covariates were adjusted.

Model 2: age and gender were adjusted.

Model 3: age, gender, body mass index, data release cycle, education level, marital status, income to poverty ratio, alcohol use, hypertension, diabetes, cancer, and cardiovascular diseases were adjusted.

© 2025 He J. et al.
